# Proteomic Profiling of Celiac-Toxic Motifs and Allergens in Cereals Containing Gluten

**DOI:** 10.1021/acs.jproteome.3c00456

**Published:** 2025-04-15

**Authors:** Matthew E. Daly, Xin Huang, Chiara Nitride, Christopher Hughes, Jaakko Tanskanen, Peter R. Shewry, Lee A. Gethings, E. N. Clare Mills

**Affiliations:** † Manchester Institute of Biotechnology, School of Biological Sciences, Manchester Academic Health Sciences Centre, The University of Manchester, Princess Street, Manchester M1 7DN, U.K.; ‡ Department of Food and Nutrition, University of Helsinki, Agnes Sjöberginkatu 2, PL 66, Helsinki FI-00014, Finland; § Department of Agricultural Sciences, University of Naples Federico II, Portici 80055, Italy; ∥ Waters Corporation, Stamford Avenue, Wilmslow SK9 4AX, U.K.; ⊥ Natural Resources Institute (LUKE), Viikinkaari 1, Helsinki 00710, Finland; # Rothamsted Research, Harpenden, Herts AL5 2JQ, U.K.; ∇ School of School of Biosciences and Medicine, The University of Surrey, Guildford GU2 7XH, U.K.

**Keywords:** Proteomics, wheat, barley, rye, oats, celiac-toxic motif, IgE epitope

## Abstract

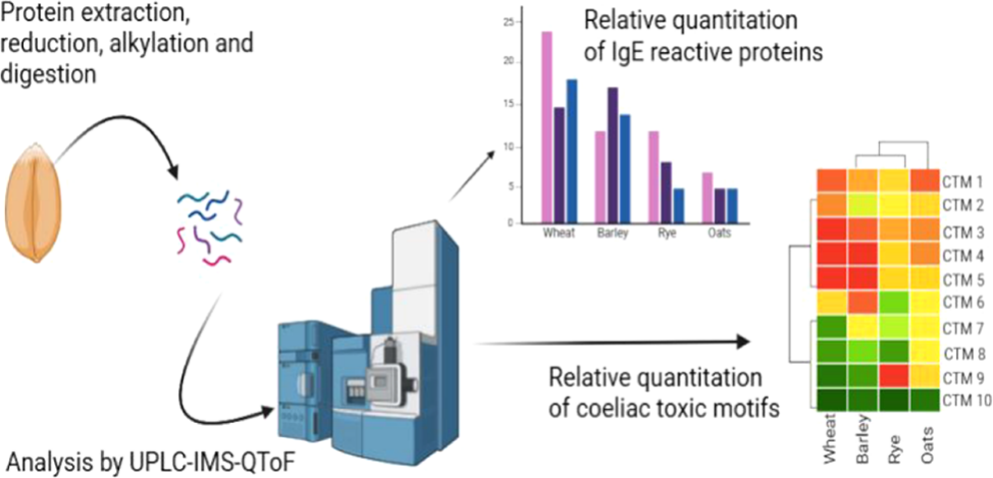

Cereal-based foods can cause immune-mediated adverse reactions, including celiac disease and IgE-mediated allergies, but the potency of different cereal species to cause such reactions appears to vary, with oats being less celiac-toxic and allergenic than wheat. In order to define differences in the immunological potential of wheat, barley, rye, and oats, proteomic profiling of proteins carrying celiac-toxic motifs and allergens has been undertaken. Total protein extracts were subjected to chymotryptic digestion and analyzed using data-independent ion mobility mass spectrometry and a pipeline employing a curated gluten protein sequence database. Depending on the cereal species, 376–2769 proteins were identified, the majority being grain storage proteins. Relative quantitation of proteins containing celiac-toxic motifs showed that they were most abundant and diverse in wheat, with only a limited number, at much lower abundance, identified in oats. Allergens belonging to the seed storage prolamins were the most abundant, while allergens belonging to the α-amylase/trypsin inhibitor family associated with respiratory allergy were of only moderate abundance in comparison. Wheat allergen homologues were identified in other cereal species but at a very low level in oats. These data suggest that the relative risk of oats in the context of both celiac disease and IgE-mediated allergy is low.

## Introduction

Wheat, barley, rye, and oats are important food crops with a combined production of ∼958 million tons in 2020,^1^ with wheat (the third-largest crop produced globally) accounting for 79.4% of the total. This scale of production of wheat reflects its unique processing properties, which allow the production of bread and other processed foods and result from the unique properties conferred by the gluten protein fraction, a combination of elasticity and viscous flow.^2^ Gluten corresponds to the seed storage prolamins, which have unusual amino acid compositions, being abundant in the amino acids proline and glutamine. They comprise a complex polymorphic mixture which is traditionally divided into two types, monomeric gliadins and polymeric glutenins.^3^ The glutenin subunits and gliadin monomers are related structurally and can be further divided into groups based on their electrophoretic mobility at low pH, sequence similarity, and the content of sulfur-containing amino acids. The grains of related cereal species, barley, rye, and oats, contain proteins homologous to wheat prolamins, known respectively as hordeins, secalins, and avenins, although the latter constitute only a minor fraction in oats.

The same proteins stimulate immune-mediated adverse reactions, including the T-cell-mediated condition known as celiac disease (CD), which can take several hours to present, and IgE-mediated hypersensitivity reactions, which present much more rapidly within less than 2 h. CD is estimated to affect up to 1% of the global population^4^ and is triggered by digestion-resistant, glutamine-rich gluten peptides, which are taken up by the gut epithelium, where the glutamine residues are deamidated by tissue transglutaminase.^5^ These deamidated peptides then bind to Human Leukocyte Antigen (HLA) class II receptor haplotypes DQ2 and DQ8, which genetically predispose an individual to celiac disease.^6^ Once presented by HLA DQ2 or DQ8, these peptides activate gluten-specific CD4+ T cells, resulting in the release of proinflammatory cytokines, with the resulting inflammatory reaction leading to flattening of the gut mucosa. These changes reduce the capacity of the gut epithelium to take up nutrients, leading to nutritional deficiencies and the characteristic “failure to thrive” symptom of celiac disease seen in children.^7^ There are extensive sequence homologies between seed storage prolamins of wheat, barley, rye, and oats, and they all carry the T-cell epitopes, also known as celiac-toxic motifs, capable of triggering CD, although the wider repertoire of prolamins in wheat means this cereal species carries the greatest burden of toxic motifs.^8,9^

With regard to IgE-mediated allergies, allergic individuals mount a specific IgE response toward cereal proteins and then experience an allergic reaction, with symptoms ranging from skin rashes to asthma, vomiting, and diarrhea. One particular type of allergy is known as wheat-dependent exercise-induced anaphylaxis (WDEIA), where symptoms are only elicited if the ingestion of wheat is combined with another compounding factor such as exercise.^10^ Major wheat allergens include the seed storage prolamins, with sensitization to ω5-gliadin being associated with WDEIA.

Currently, there is no cure for either CD or IgE-mediated food allergy, and consequently, individuals with these conditions must practice lifelong avoidance of foods containing either gluten or wheat. In order to help them make safe choices, foods containing cereal ingredients from wheat, barley, rye, and oats must be labeled as cereals containing gluten, as defined by the Codex Alimentarius Commission, although oats are not considered to contain gluten as specified by the Food and Drug Administration (FDA) of the USA, unlike the European Union and the UK.^11^ Foods containing <20 mg/kg gluten can carry a “gluten-free” claim,^12^ while precautionary allergen labels are often used to inform consumers about possible unintended allergen presence that results from agricultural commingling or the use of shared processing equipment and facilities.^13^ One approach to harmonize their use is to apply risk-based approaches,^14^ where action levels are calculated from reference doses of allergenic food proteins identified in clinical food challenge studies.^15^ These doses indicate the potency of a food to cause an adverse reaction, which is in turn determined by the profile of food ingredient proteins that carry either celiac-toxic motifs or allergen molecules. The levels of celiac-toxic motifs and allergens in a given food product may change as a consequence of breeding or as a result of food processing. Therefore, understanding the relative potency of different cereal species in terms of their ability to cause CD or IgE-mediated food allergies is important to benchmark, in order to allow any changes in potency that may result from shifts in molecular profiles to be identified over time.

Proteomics provides a platform for the characterization of the total proteome of an organism, and mass spectrometry (MS) analysis allows the identification and quantitation of celiac-toxic motif-containing proteins and allergens. These approaches therefore have great potential to monitor the allergenic potency of foods and have previously been applied to the identification and relative quantitation of immunoreactive proteins in wheat, barley, and rye,^16^ relative quantitation of IgE epitopes and celiac-toxic motifs in wheat and different wheat species,^17^ absolute quantitation of the canonical immunodominant sequence associated with celiac disease (33mer) in wheat,^18^ and immunotoxic epitope mapping of peptides in wheat, barley, and rye.^19^ Further, identification of common proteins between two wheat cultivars for the purpose of targeting high-heritability proteins has also been undertaken.^20^ Mapping of predicted gene products to those observed from proteomics has also been applied to wheat,^21^ with a focus on allergens.^22^ However, a direct comparison of the seed storage prolamins of different cereal species and the associated burden of celiac-toxic motifs and IgE epitopes using proteomics approaches has not been previously undertaken. Therefore, proteome profiling of “cereals containing gluten” (bread wheat (*Triticum aestivum*), barley (*Hordeum vulgare*), rye (*Secale cereale*), and oats (*Avena sativa*)) has been undertaken using MS and applying an analysis pipeline utilizing curated sequence databases for seed storage prolamins, IgE-mediated food allergens, celiac-toxic motifs, and IgE-epitopes.^8^ This has allowed us to test the premise that the relative abundances of celiac-toxic motifs and IgE epitopes vary between different cereal species to be defined and that the burden of toxic motifs is lower in oats, which explains the observation that many patients with CD or IgE-mediated food allergies can tolerate them.

## Experimental Section

### Materials

All reagents used were of analytical grade unless stated otherwise. Wholemeal flour from wheat (*Triticum aestivum* cv Chinese Spring) was provided by Rothamsted Research (Harpenden, UK), grains of barley (*Hordeum vulgare* cv Morex) and oats (*Avena sativa* cv Aslak) were provided by the Natural Resources Institute Finland (LUKE) (Helsinki, Finland), and rye grain (*Secale cereale* inbred cv Lo7) were obtained from KWS LOCHOW (Bergen, Germany). These cultivars were chosen because of the availability of genomic or transcriptome data, with the bread wheat cv Chinese Spring,^23^ the six-row malting barley cv Morex,^24^ and the inbred rye line Lo7^25^ being cultivars chosen to form the annotated reference genomes of those cereals, while the hexaploid oat cv Aslak is one that is included in the ongoing Oat Pangenome project. Formic acid, acetonitrile, and water used in chromatography were all HPLC grade (Sigma-Aldrich, Dorset, UK). Formic acid, acetonitrile, and water used for LC–MS were all LC–MS grade (Sigma-Aldrich, Dorset, UK). Urea, ethanol, propan-2-ol, tris(hydroxymethyl)aminomethane, dithiothreitol (DTT), iodoacetamide, and enolase from baker’s yeast (*Saccharomyces cerevisiae*) were purchased from Sigma-Aldrich (Poole, Dorset, UK). Low-binding microcentrifuge tubes were obtained from Sarstedt (Nümbrecht, Germany). Sequencing-grade chymotrypsin (Promega, Madison, USA) from porcine pancreas, with an activity of >70 U/mg (measured by benzoyl-L-tyrosine ethyl ester (BTEE) assay), was used for proteolysis. RapiGest (sodium 3-[(2-methyl-2-undecyl-1,3-dioxolan-4-yl)methoxy]-1-propanesulfonate) was obtained from Waters Corporation (Milford, MA, USA). NuPAGE Bis-Tris gels (4–12%), NuPAGE lithium dodecyl sulfate (LDS) buffer (4×, pH 8.4), 2-(*N*-morpholino)ethanesulfonic acid (MES) buffer (20× concentrate), SYPRO Ruby Protein Gel Stain, and Mark 12 protein markers were purchased from Invitrogen (Shropshire, UK).

### Sample Preparation, Extraction, and Quantitation

Whole seeds of the barley, rye, and oat cultivars were milled using a consumer coffee grinder (Maison and White, Oxford, UK) and further ground using a pestle and mortar prior to extraction. Three samples of either ground seed or flour (50 mg) were extracted with 1 mL of 50% (v:v) propan-2-ol containing 100 mM Tris-HCl, pH 7.5, 2 M urea, and 60 mM DTT at 60 °C with sonication for 15 min and vortexing every 5 min (three biological replicates per cereal). Extracts were clarified by centrifugation for 10 min at 10,000*g,* and supernatants were collected and transferred to fresh microcentrifuge tubes.^26^ Protein content was determined in duplicate using the RC DC Protein Assay (Bio-Rad, Watford, Hertfordshire, UK), using bovine serum albumin for the calibration curve at 0, 0.125, 0.25, 0.5, 1, and 2 mg/mL. This is a modified Lowry protein assay^27^ and allows quantitation of protein in the presence of reducing agents and detergents. Briefly, proteins present in solution were precipitated, and any interfering substances were removed after centrifugation. After resuspension, alkaline copper tartrate solution and Folin’s reagent were added, and the absorbance was read at 750 nm using a Biochrom Asys UVM-340 (Cambridge, UK) microplate reader.

### Reduction, Alkylation, Digestion, and Sample Cleanup

Enolase from baker’s yeast (*S. cerevisiae*) was included as a digestion control; a 1 mg/mL enolase stock solution was prepared in 100 mM Tris-HCl pH 7.5, and disulfide bonds were reduced by the addition of DTT to a final concentration of 60 mM, followed by heating to 80 °C for 10 min. The enolase was added to the grain extracts to give a final concentration of 10 μg/mL. Cysteine residues in the reduced samples were alkylated by the addition of iodoacetamide to give >2:1 molar excess over DTT (final concentration; 40 mM DTT and 100 mM iodoacetamide) and incubated in the dark for 30 min. Reduced and alkylated samples were then diluted 1:5 (v:v) with 100 mM Tris-HCl, pH 8.0, containing 10 mM CaCl_2_ and 0.1% (w/v) RapiGest (final concentrations) to give a final propan-2-ol level of <10% and a urea concentration of <0.4 M, so as not to interfere with protein digestion. Diluted samples were placed in low-binding microcentrifuge tubes (Sarstedt, Nümbrecht, Germany), and chymotrypsin from porcine pancreas was added at a 1:100 (w:w) protease:protein ratio and incubated for 18 h at 37 °C in a Stuart SBS40 (Cole-Palmer, St Neots, UK) shaking water bath. After 18 h, digestion was stopped by the addition of formic acid to a final concentration of 0.1% (v:v), samples were clarified by centrifugation for 10 min at 10,000*g,* and supernatants were transferred into fresh low-binding microcentrifuge tubes. Samples were desalted by solid-phase extraction (SPE) using Sep-Pak C18 Vac cartridges according to the manufacturer’s instructions (Waters, Wilmslow, UK) and concentrated using an Eppendorf Concentrator Plus (Stevenage, UK) to a final protein concentration of 250 μg/mL, based on the starting protein concentration calculated using the RC DC Protein Assay (Bio-Rad, Watford, Hertfordshire, UK). The effectiveness of the digestion protocol was checked using SDS-PAGE and HPLC (Supporting Information S1).

### Liquid Chromatography–Ion Mobility Mass Spectrometry

Digested, cleaned-up samples were then analyzed by liquid chromatography–ion mobility mass spectrometry (LC–IM-MS). Two hundred and fifty nanoliters of each sample was injected and chromatographically separated using reversed-phase chromatography and an ACQUITY M-Class (Waters, Milford, USA) configured in Trap and Elute mode. Each biological replicate was injected three times (technical replicates), leading to nine data files per cereal. Solvent A was 0.1% (v/v) aqueous formic acid, and solvent B was acetonitrile containing 0.1% (v/v) formic acid. The trapping column was a 180 μm × 20 mm, Symmetry C18, 100 Å, 5 μm (Waters, Wilmslow, UK), and the analytical column was a 75 μm × 250 mm, HSST3 C18, 100 Å, 1.8 μm (Waters, Wilmslow, UK). Trapping was carried out at 5 μL/min for 2 min at 99% solvent A, before the peptides were eluted from the column at 5–40% solvent B over 90 min, followed by a wash at 85% solvent B and column re-equilibration at 5% solvent B for a further 30 min at 300 nL/min. The eluate was directed into a SYNAPT XS (Waters, Wilmslow, UK), and data were acquired using ion mobility-enabled MS^E^ (High Definition MS^E^, HDMS^E^) mode and a data-independent acquisition (DIA) in positive ion mode over the mass range *m*/*z* of 50–2000 with a 0.5 s spectral acquisition time, providing one cycle of low and elevated energy data every 1 s to gain information about precursor and fragment ions, respectively. A collision energy profile was applied using a lookup table: 0.7 to 85 V over 195 mobility “bins”. The reference LockSpray used a solution of Glu1-Fibrinopeptide, which was infused at 1 μL/min, and sampled every 2 min. Data were collected over 115 min using MassLynx version 4.1 (Waters, Wilmslow, UK). Data are available via ProteomeXchange with the identifier PXD039539.

### Mass Spectrometry Data Analysis

Raw files obtained from the LC–IM-MS acquisitions were imported into Progenesis QI for Proteomics (version 8.0) on a species-specific basis. Data were processed using Waters algorithms (Apex3D64 and Peptide3D) and analyzed using the Ion Accounting workflow that is optimized for processing ion mobility DIA data with a low intensity threshold set to 150 counts and a high intensity threshold set to 30 counts.^28^ Imported data sets were then searched against the total sequence set attributed to the “Viridiplantae” taxonomy available in UniProt (9576972 sequences; accessed 14.04.2020), appended with the common Repository of Adventitious Proteins (cRAP) database, as well as sequences for porcine chymotrypsin and enolase from baker’s yeast, with the combined database split into 10 roughly equal subdatabases to facilitate analysis. Data were searched against each subdatabase individually and then recombined within Progenesis QIP using the “Recombine Fractions” functionality. The protease was set to chymotrypsin with cleavage at tyrosine, phenylalanine, tryptophan, or leucine unless followed by a proline, with up to two missed cleavages. Fixed modifications were specified as carbamidomethylation of cysteine, and variable modifications were set to hydroxylation of proline, oxidation of methionine, deamidation of glutamine or asparagine, and finally, N-pyroglutamic acid formation from either glutamine or glutamic acid. The false discovery rate (FDR) was set to 1%, and mass tolerance for peptide and fragment ions was set to 10 and 20 ppm, respectively. Peptides identified in only one technical replicate of each biological replicate and/or with a peptide score <5 were removed. A well-defined data analysis and curation pipeline was created and implemented for this study (Figure S5). Extracted ion chromatograms for two exemplar peptides, one with a high peptide score and one with a low peptide score, identified during searching are presented in Figure S6. Where proteins were identified with only one unique peptide, the extracted ion chromatograms for that peptide were reviewed, and those where fragment ions were not clearly identified were removed. Following initial searching against a database containing all proteins from species with the taxonomy set to Viridiplantae in UniProt, protein IDs were further filtered to contain only those found in either the reference sequenced genomes of relevant cereal species (wheat cv Chinese Spring (UP000019116); barley cv Morex (UP000011116); rye Lo7 (GCA_902687465)) or present in the GluPro suite of curated gluten sequences.^8^ Peptide raw abundances were renormalized after output from Progenesis QIP to the raw abundance of the yeast enolase peptide DSRGNPTVEVELTTEKGVF, as this was the most intense peptide identified for this protein across runs.

### In Silico Analysis of Protein Profiles, Celiac-Toxic Motifs, and IgE Epitopes

A variety of *in silico* tools were used to analyze the data generated during the mass spectrometry analysis. MetaboAnalyst 5.0 (https://www.metaboanalyst.ca/)^29^ was used for multivariate statistics and the generation of 3D PCA scores and loadings plots. Data were scaled using Pareto scaling and log transformed prior to the generation of the PCA and loadings plots. This platform was also used to generate heat maps of the abundance of celiac-toxic motif-containing peptides across the cereal grains identified in mass spectrometry. Gene Ontology terms were searched using QuickGO browser,^30^ and results were downloaded in CSV format. Phylogenetic trees were generated using Jalview^31^ and edited using FigTree v1.4.3, as described previously.^8^ Identification of allergen homologues employed many different tools. Initially, whole sequence BLAST was conducted on UniProt (UniProt.org; accessed 7/7/2022),^32^ whereas Python (Python Software Foundation, Python Language Reference, version 3.8, available at http://www.python.org) was used to section each protein sequence into sequences of 80 amino acids with a step of one. These 80-mer sequences were then searched using the FASTA algorithm^33^ and a custom R v4.0.2 script^34^ that collated the results and reported how many hits with an identity >35% were identified for that protein. Homologues identified that were partial sequences were discarded. A database comprising 1,041 CD-active peptides was downloaded from AllergenOnline (AllergenOnline.com; accessed 21/06/2022),^35^ and peptides identified from mass spectrometry containing any of these CD-active peptides were collated, their abundance summed using Microsoft Excel (Microsoft Corporation, 2018), and the resulting data were visualized using MetaboAnalyst. All graphs displayed in the manuscript were generated using GraphPad Prism 8 for Windows (GraphPad Software, San Diego, CA, USA).

## Results and Discussion

### Proteomic Profiling of Grains from Cereal Species Containing Gluten

Initially, the quality of the sample preparation and digestion was assessed using 1D-PAGE and RP-HPLC (Supporting Information 1), which demonstrated the absence of large polypeptides and a clearly different HPLC profile, indicating sufficient digestion of the gluten proteins.

Data were searched against the entire Viridiplantae database to identify the proteomes of wheat, barley, rye, and oat grains (Supplementary Data files 1, 2, and 4). A protein was considered identified if at least one unique peptide was identified for that protein accession in at least two technical replicates of each biological replicate analyzed, with a peptide score >5. However, this approach excludes proteins, which are highly homologous, have repetitive sequences (such as the cereal seed storage prolamins), and lack a unique chymotryptic peptide. This limitation can be overcome using protein grouping, where proteins with common peptides unique to the group can be classified under one “lead” accession. However, this does not guarantee that all proteins listed within the group are present in the sample. Following this approach, the largest number of protein groups was identified for rye (3,211), where 2,769 protein accessions were identified with a unique peptide, followed by barley and wheat (with 1,996 and 1,553 groups and 1,700 and 1,453 proteins with unique peptides, respectively), with the fewest groups being identified for oats (with 409 groups and 376 protein accessions with a unique peptide) (Table 1). The greater diversity in rye likely reflects its being more polymorphic as a result of its outbreeding nature, with wheat being similar despite the hexaploid nature of wheat. Indeed, others have suggested that the relationship between ploidy level and proteome complexity is not straightforward due to nonadditive gene expression^36^ although proteins are known to be expressed on all three genomes in wheat. Gene ontology (GO) analysis of the identified proteins showed that the majority had nutrient reservoir activity (Figure S2), with the majority of which being seed storage proteins comprising either seed storage prolamins or, in oats, seed storage globulins.

**Table 1:** Proteomic Profiling of Cereal Species Containing Gluten^a^

	Wheat (*T. aestivum*)		Barley (*H. vulgare*)		Rye (*S. cereale*)		Oats (*A. sativa*)	
Search strategy	Protein grouping	Protein	Protein grouping	Protein	Protein grouping	Protein	Protein grouping	Protein
No of sequences identified in Viridiplantae	1,553	1,453	1,996	1,700	3,211	2,769	409	376
Identifications filtered by relevant species	341	321	278	239	36	33	35	30
Identifications filtered by relevant species genome or transcriptome.	155	149	42	61	36	4	10	6
Identifications filtered by relevant species GluPro database	93	85	28	31	9	9	9	8

a Protein – protein groups identified from Progenesis searches; Peptide – proteins identified with a unique peptide without protein grouping, i.e., identification achieved with protein grouping off. UniProt searching was filtered by taxonomy “Viridiplantae” (*n* = 9,576,972 protein sequence accessions), and sequences filtered based on either assignment to a particular cereal species sequences, sequences present in translated cDNA (wheat cv Chinese Spring, IWGSC, INSDC Assembly GCA_900519105, *n* = 107,891); barley cv Morex (INSDC Assembly GCA_903813605.1, *n* = 32,159), and *A. sativa* (alignments available; 10.6084/m9.figshare.25672209), or mRNA (*S. cereale* rye Lo7 (GCA_902687465, *n* = 34,441), or assignment in the curated GluPro database.^8^

The application of data-independent acquisition (DIA) was selected for this analysis combined with ion mobility due to the inherent unbiased sampling of the MS1 of DIA while also increasing the peak capacity due to the ion mobility dimension. It has been demonstrated that profiling wheat grains using IMS-DIA resulted in a higher number of peptide identifications compared to a data-dependent acquisition (DDA) completed on a linear ion trap.^37^

**Figure 1:**
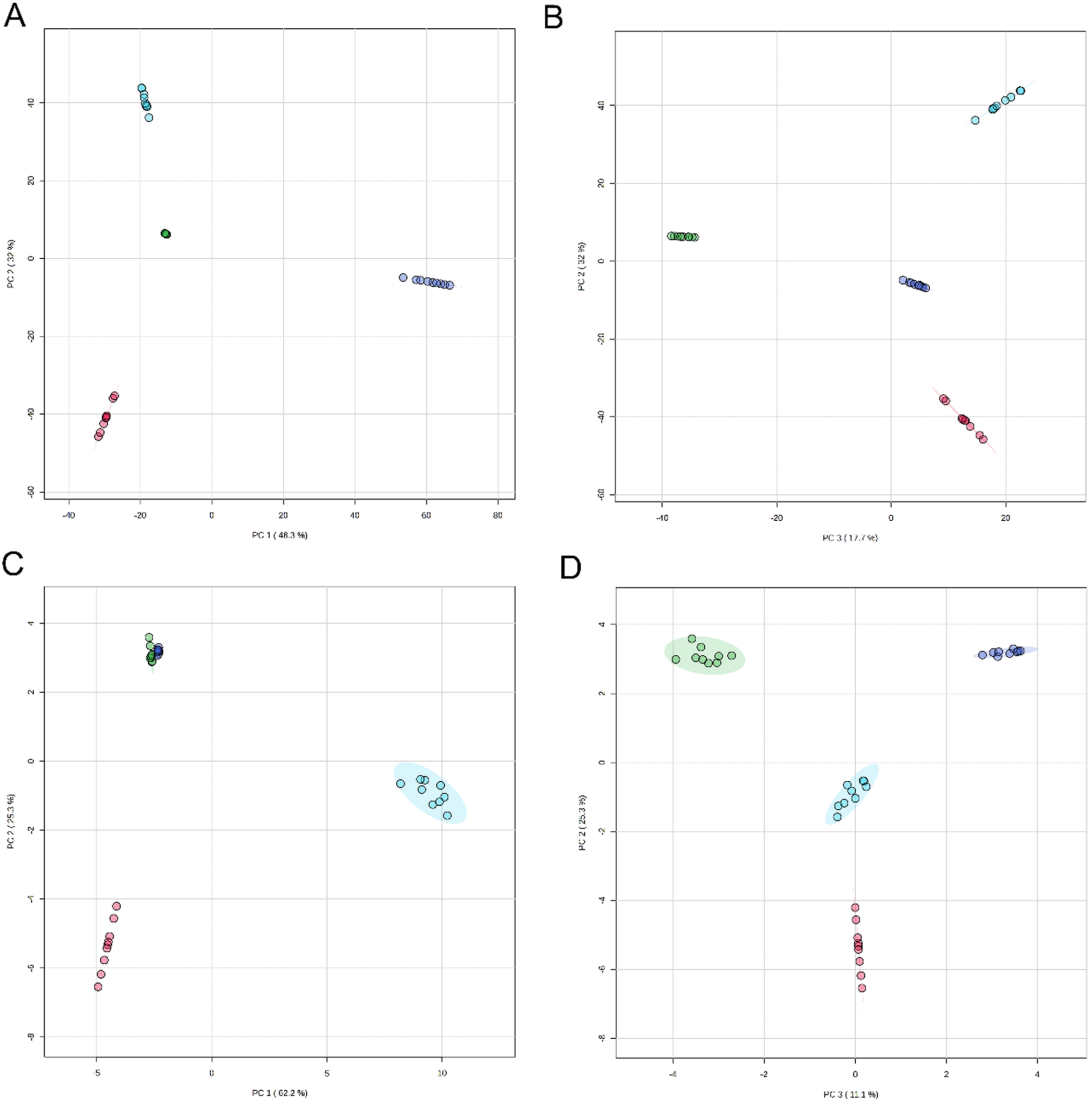
Principal component analysis (PCA) of proteome profiles of wheat, barley, rye, and oats. PCA scores plot of UniProt protein accessions (lead accession from protein grouping) relative normalized abundances identified in wheat (*T. aestivum*; light blue circles), barley (*H. vulgare*; red circles), rye (*S. cereale*; dark blue circles), and oat (*A. sativa*; green circles) when searching against *Viridiplantae* (A; PC1 vs PC2, B: PC2 vs PC3) and tagging accessions identified in GluPro v 6.1 C; PC1 vs PC2, D; PC2 vs PC3). 3D loading plots are available in Figure S5 and Supporting Information Data File 3 -– Sheet 1.

Principal component analysis (PCA) of the proteomics profiles allowed the classification of different cereal species (Figure 1A), with the first three components accounting for 99.3% of the variance observed. Loadings plots showed that three of the five proteins with the largest positive value contributions to PC1 were γ-type prolamins (Figure S1 and Supplementary Data File 3 - Sheet 1). However, the majority of the proteins contributing to the loadings were not prolamins and included nonspecific lipid transfer proteins (Table S1). This is consistent with the known high levels of homology between seed storage prolamins in wheat, barley, rye, and oats,^8^ with the greater diversity in nongluten protein analysis presenting better targets for differentiation between cereal species.

### Proteomic Profiling of Gluten Proteins

**Figure 2:**
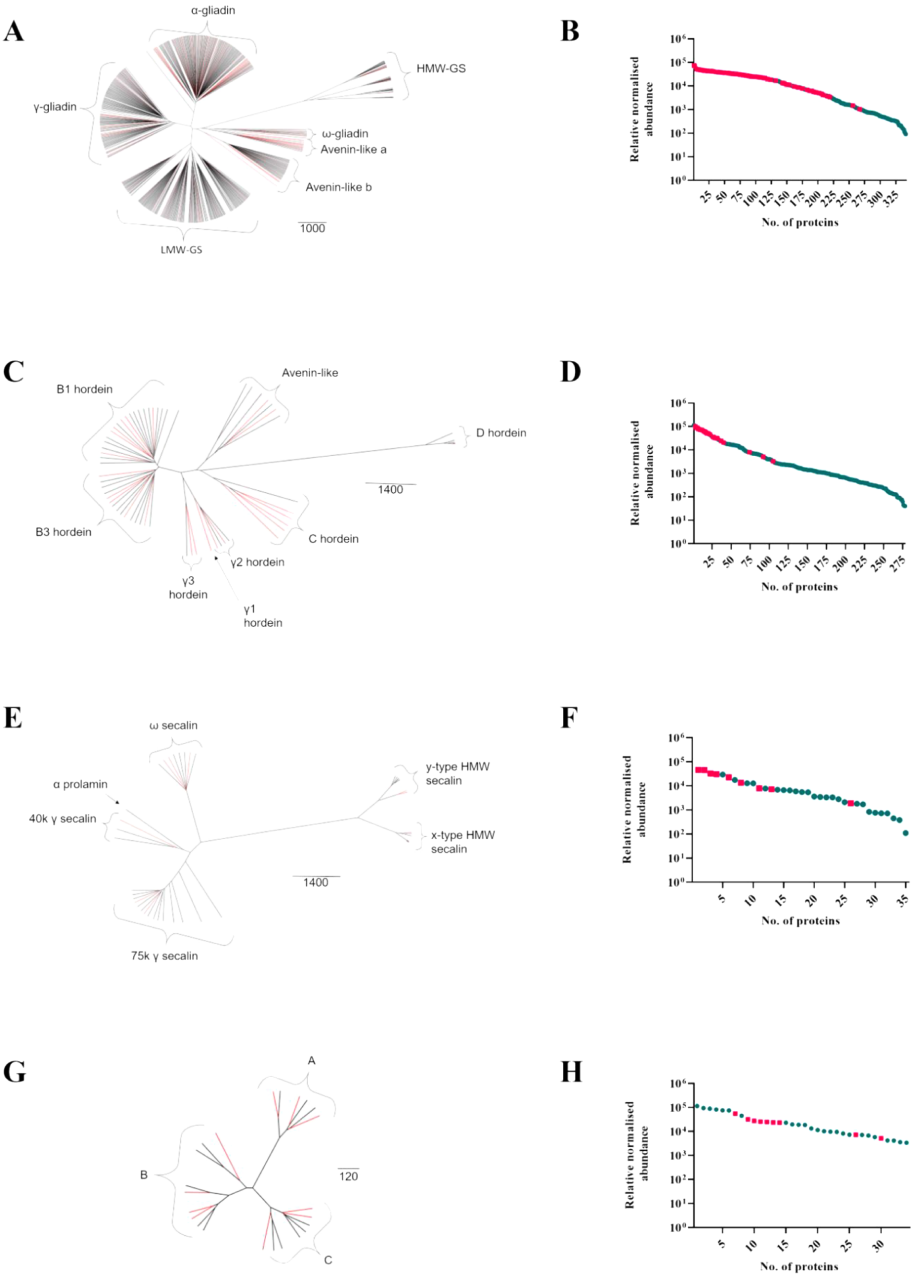
Phylogenetic analysis and relative quantification of identified gluten proteins. Protein accessions tagged as gluten proteins in the GluPro curated gluten protein sequence databases^8^ were mapped either into the relevant GluPro phylogenetic tree (A, C, E, G) or Quant curves (B, D, F, H), where GluPro identifications were colored in pink and the total species specific identifications shown in green. (A, B) wheat—GluPro v 1.2; (C, D) barley—GluPro v 3; (E, F) rye—GluPro v 4; and (G, H) oats—GluPro v 5 (G). Protein accessions used to generate the figures were the lead accessions from protein grouping.

Total proteome data was further analyzed using the GluPro suite of databases containing curated gluten proteins from *T. aestivum*, *H. vulgare*, *S. cereale*, and *A. sativa* (Table 1), with similar numbers of annotations being made compared to other studies focusing on gluten protein analysis from mature wheat grain.^21,22^ Mapping onto the GluPro phylogenetic trees showed the wide and complete identification of protein sequences from all gluten protein types in all species analyzed (Figure 2A, C, E, and G). This is an improvement compared to previous analysis,^8^ which may be due to the use of an optimized extraction and sample preparation procedure.^26^ Specific protein isoforms known to be present in the wheat cv Chinese Spring were not identified on the basis of unique peptides but could be identified using protein grouping on the basis of common peptides. For example, the 1Dx subunit will never be reliably identified using mass spectrometry due to the lack of unique peptides generated by chymotrypsin, but peptides corresponding to the 1Dx subunit type were identified.^38^ In addition, although unique chymotryptic peptides for the high molecular weight glutenin subunits (HMW-GS) 1By and 1Bx can be predicted from the wheat cv Chinese Spring genome, they are mostly based on missed cleavages, are long, and encompass the same region of the sequence and so may not be routinely observed using a standard chymotryptic workflow.

The relative abundances of the different seed storage prolamins identified were then considered within the overall range of relative protein abundances for species-specific accessions (Figure 2B, D, F, and H). They dominated the abundance profiles of all cereal species except oats, for which the avenins were ranked in the middle order, the most abundant proteins identified being the seed storage globulins.^39^ This reflects the relatively low abundance of avenins in oats, which comprise only about 10–15% of total seed proteins, and high abundance of storage globulins, which comprise about 50% of total seed proteins.^40^ The abundance spanned 3 orders of magnitude, showing that certain prolamin proteins are very minor components. PCA analysis of seed storage prolamins from the different species showed that 98.6% of the variance was accounted for in the first three components (Figure 1B). Loading values for the PCA showed that the major proteins contributing to the separations in both PC1 and PC2 were from *H. vulgare* (Table S2 and Supporting Information File 3 – Sheet 2). *Avena sativa* proteins were responsible for the separation in PC3, particularly in the negative direction.

### Identification and Relative Abundances of Peptides Containing Celiac-Toxic Motifs in Cereal Species

**Figure 3:**
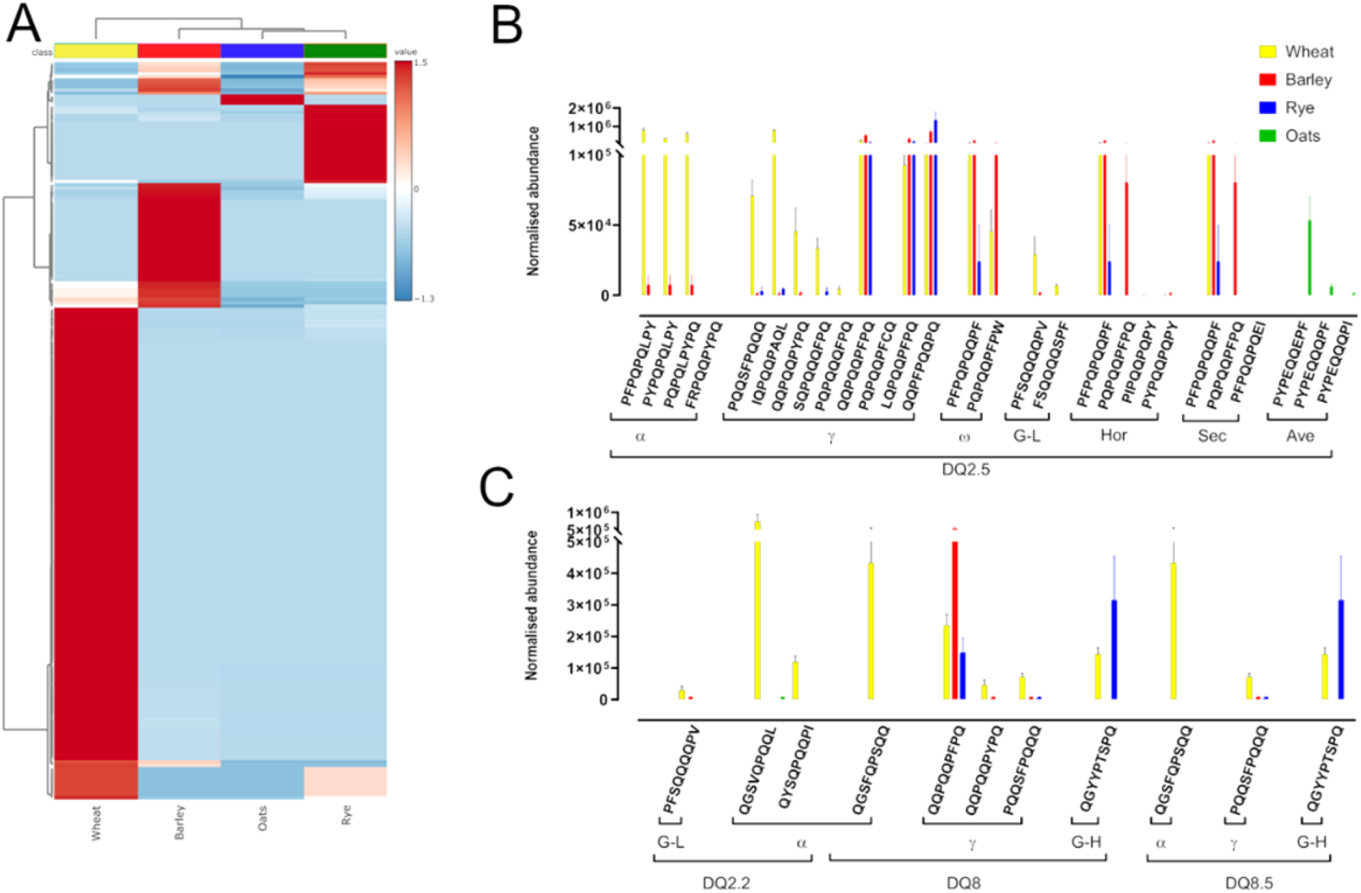
Distribution of celiac-toxic motifs in cereal seed proteomes. (A) Heat map of celiac-toxic motif-containing peptides retrieved from AllergenOnline and identified from discovery mass spectrometry (auto-scaled to features). Columns are specified by the classes, which were as follows: wheat—yellow; barley—red; rye—dark blue; oatsgreen. Relative normalized abundance of consensus celiac-toxic epitopes for DQ2.5 (B) and DQ2.2, DQ8 and DQ8.5 (C)^5^ α—α-gliadin; γ—γ-gliadin; ω—ω-gliadin; G-LLMW-GS; Horhordein, Sec—secalin; Ave—avenin; G-HHMW-GS. Cereal species are denoted by colored bars as in panel A. It was also clear that some peptides containing CTMs were present in combinations of wheat, barley, and rye, with varying abundances, but none were common to all three cereal species.

The normalized abundances of peptides containing celiac-toxic motifs (CTMs), corresponding to epitopes able to activate T-cells in individuals with celiac disease, were analyzed using the 1041 CD-active peptides deposited in AllergenOnline.^35^ Specifically, only peptides and their associated normalized abundance identified during mass spectrometry analysis that totally encompassed a CD-active peptide, as defined in AllergenOnline, were considered as containing T-cell epitopes. Only 225 peptides containing CTMs were found (Supplementary Data File 6), and heat map analysis revealed distinct differences in the patterns and abundances of peptides across the different cereal species (Figure 3A). Cluster analysis showed that wheat was separated from the other cereal species, with rye and oats containing both lower amounts and less diverse CTMs than wheat or barley. Wheat contained the largest number and diversity, confirming previous *in silico* analysis.^8^

**Figure 4:**
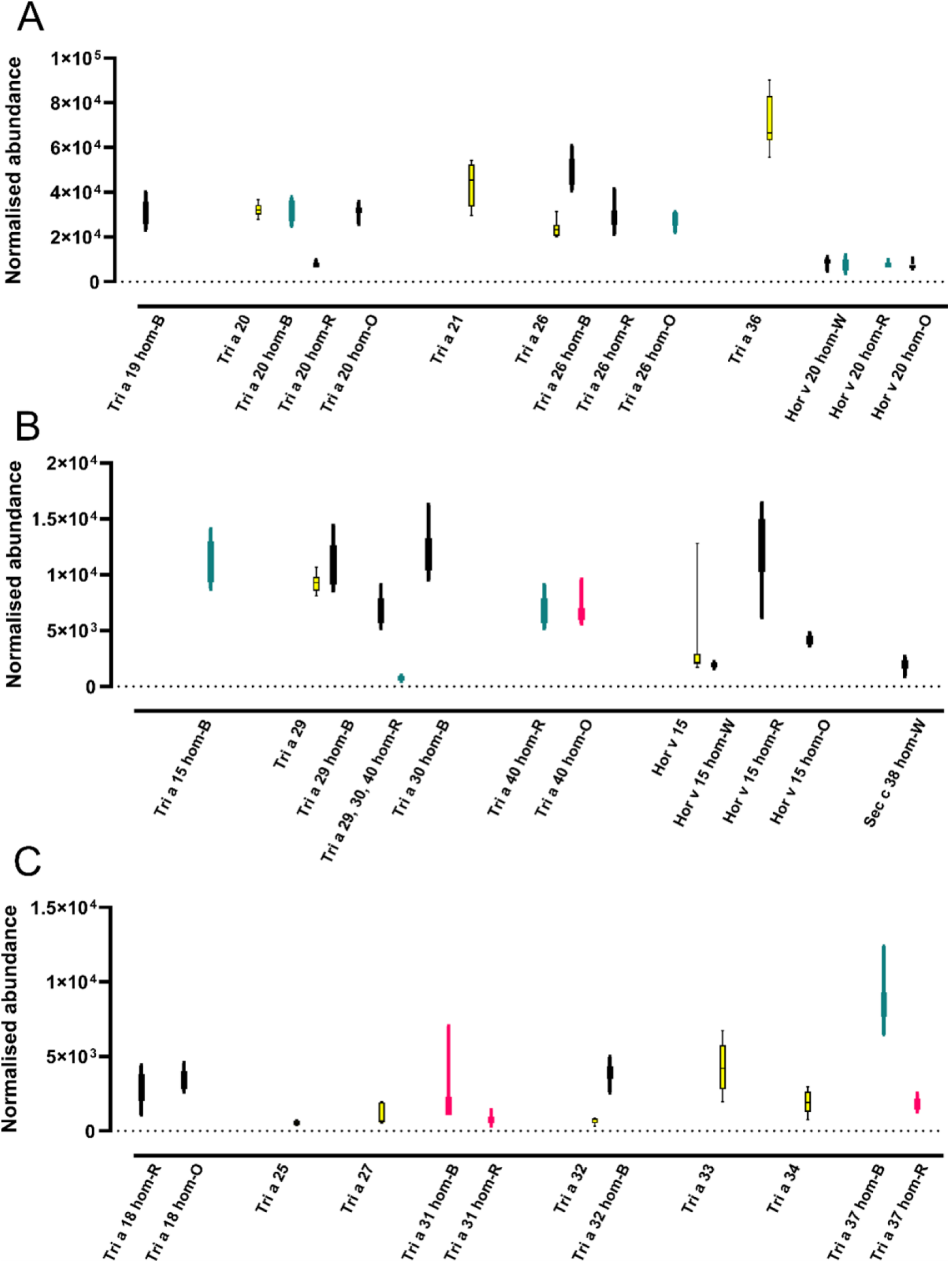
Identification and relative abundance of allergens associated with IgE-mediated allergies using the lead accession from the protein grouping. Yellow bars indicate the WHO allergen isoform, black bars are homologues identified through full sequence BLAST, green bars are those identified from a sliding 80mer window and FASTA, and pink bars are the accessions that were common between the two methods. Allergen sequences were retrieved from the WHO/IUIS allergen nomenclature database and homologues in other cereal species identified from discovery mass spectrometry. Allergens were grouped as being either gluten proteins (A), trypsin/α-amylase inhibitors (ATI) (B), or other (C). Homologues are identified as follows: wheat—Hom-W; barley—hom-B; rye—hom-R; oats—hom-O.

Peptides containing CTMs identified in oats were all unique to that species, reflecting the species-specific nature of the B-type avenins. Additional analysis was also performed using the consensus T-cell-restricted epitopes identified by Sollid and coworkers (Figure 4B). Thirty-three of the 38 epitopes were found in the cereal proteomes, with those not identified all being DQ2.5 restricted. Three of the epitopes not identified span chymotryptic digestion sites and would only be identified, which explains their absence from the data sets. The two other “missing” epitopes, one each from a secalin and an avenin, are either of very low abundance or absent from the cultivars used in this analysis. Some T-cell epitopes classified as “Hor” (hordein) and “Sec” (secalin) by Sollid et al. were found in wheat, barley, and rye, and were least abundant in rye. Of the different cereal species, wheat contained the highest abundance of T-cell epitopes, mainly due to those present in the α-gliadin fraction, which is both abundant and only present in wheat. DQ-restricted epitopes were also very abundant in the γ-type prolamins, with barley containing the highest abundance of two epitopes (QQPQQPFPQ and LQPQQPFPQ) also found in wheat, barley, and rye. Of the remaining DQ-restricted epitopes in γ-type prolamins, one shared across all cereal species (QQPFPQQPQ) was most abundant in rye, with the remaining being most abundant in wheat. The T-cell epitopes originating from ω-type prolamins, one of which is considered to be one of the most potent T-cell stimulatory peptides in CD,^41^ were less abundant, reflecting the lower abundance of this gluten protein type. The least abundant T-cell epitopes were those found in avenins, of which only two were identified at a low level and only in oats.

### Proteomic Profiling of Wheat Allergens

Allergen accessions associated with IgE-mediated food allergies were retrieved from the WHO/IUIS allergen nomenclature database, except for oats (*A. sativa*) for which no allergens are listed.^42^ These were used to identify allergens in the proteome profiles of different cereal species. Additional isoforms present in the proteomics profiles of the different cereal species that could represent cross-reactive allergens were also identified. Putative cross-reactive allergen sequences were also identified using either a full sequence BLAST or a sliding 80-mer window and FASTA (Table 2 and Tables S3 and S4, 10.6084/m9.figshare.21916854).^43^ This broad approach was chosen because it should identify allergen homologues and address criticisms of the sliding 80-mer window, which can identify proteins as potential allergens that have very low levels of homology and are unlikely to be allergenic.^44^ Based on the data analysis described above, the identified proteins in all the cereal species were investigated for the presence of these allergenic proteins, either canonical sequences or newly identified putative cross-reactive allergens (Table 2). Two rye allergens (Sec c 20 (two isoforms) and Sec c 38) and two wheat allergens (Tri a 41 and 42) were identified, which are fragments less than 80 amino acids in length and could only be analyzed using BLAST searching. There was little overlap in the accessions identified by the different methods, with only 11 homologues identified for barley, 10 for rye, and 5 for oats by both approaches. These spanned inhalant allergens from pollens such as the profilin allergen from wheat, Tri a 12, and those involved in Bakers’ asthma, such as the amylase/trypsin inhibitors (ATIs) Tri a 40 (Table S5). Interestingly using either the BLAST or FASTA methods, only two WHO/IUIS allergen isoforms (both from rye) mapped as homologues of other cereal allergens, the Phl p 5 pollen allergen family member, Sec c 5 mapping as a Hor v 5 homologue, and the γ-secalin allergen Sec c 20 mapping as a homologue of wheat ω5-gliadin (Tri a 19).

**Table 2:**
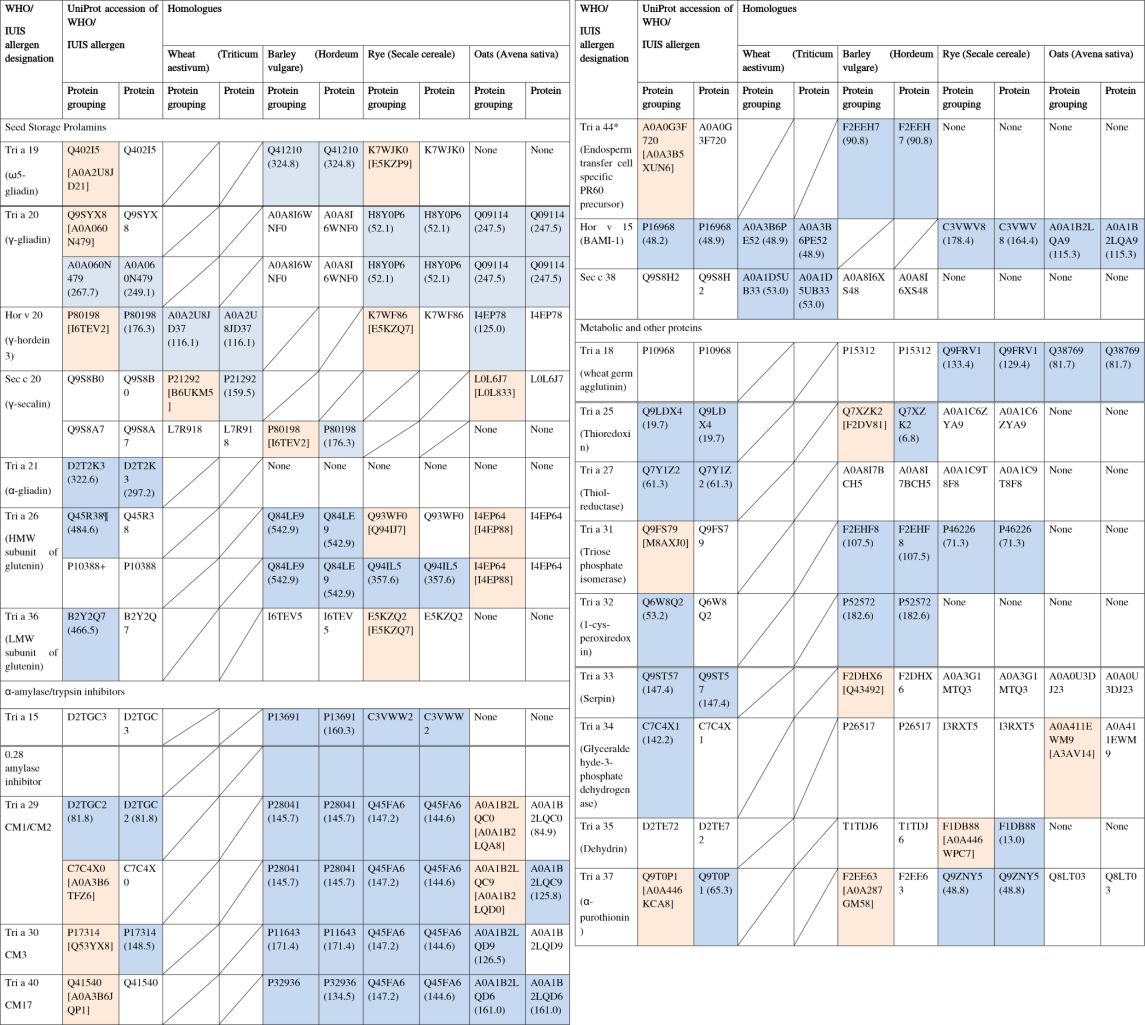
Allergen Isoforms Were Identified from Profiling Cereals Containing Gluten^a^

a Mass spectral data were searched, and allergen isoforms designated in the WHO/IUIS allergen nomenclature database or homologues were identified. Boxes shaded in blue indicate that the accession was identified in mass spectral data using either protein grouping (Protein grouping) or proteins with at least one unique peptide (Protein). The confidence score of the identified protein accession is shown within parentheses after the accession. Boxes shaded in orange indicate that the protein was identified within a protein group with the lead accession of that group shown in square brackets. The lead accession has the highest protein score and hence highest probability of being present in the sample. If the accession was not the lead accession identified using Protein Grouping, then no confidence score was assigned. * Belongs to the prolamin superfamily. ¶ corresponds to Tri a 26.0201; + corresponds to Tri a 26.0101.

The expanded sequence sets resulting from this analysis were then used to mine the proteomic profiles of each of the cereal species (Table 2). No evidence was found of the pollen profilin allergen and its homologues from wheat (Tri a 12) and barley (Hor v 12), or the pollen Phl p 5 family (Hor v 5 and Sec c 5), which is consistent with their being expressed in pollen and not cereal seed tissues. Evidence was also lacking for specific accessions attributed to the wheat LTP allergen, Tri a 14, β-amylase (Tri a 17), the 0.19 ATI (Tri a 28), the serine protease inhibitor-like protein belonging to the potato tuber inhibitor family (Tri a 39), and four wheat proteins that are involved in metabolism (Tri a 41–43 and 45). However, other isoforms of these proteins were identified (Supporting Information, Files 1 and 3). Using protein grouping and/or accessions with unique peptides identified five wheat seed storage protein allergens together with two ATIs and six proteins with other functions (Table 2). Since wheat cv Chinese Spring has the HMW-GS composition 1Bx6 + 1By8 and 1Dx2 + 1Dy12, the allergenic HMW subunit 1Dx5 (Tri a 26.0101) was not identified, but the 1Bx7 subunit (Tri a 26.0201) was, likely reflecting its homology to the 1Bx6 subunit of cv Chinese Spring. Interestingly, homologues of Tri a 26 were identified in barley and rye, suggesting that D hordeins and HMW secalins have homology with 1Dx5. Similarly, the ω5-gliadin allergen, Tri a 19, was not identified in wheat, but a homologue, C-hordein, was identified in barley. Allergenic γ-gliadins were also identified from wheat (Tri a 20), barley (Hor v 20), and rye (Sec c 20). A number of ATI allergens were identified in wheat, barley (Hor v 15), and rye (Sec c 38) together with putative homologues. Searching for proteoforms is notoriously difficult, and it is not surprising that many specific allergen sequence accessions were not identified, although homologues and closely related proteins were (Supplementary Data Files 1, 2, and 4).

The relative abundance of the cereal allergen accessions and putative cross-reactive allergens in different cereal species was determined (Figure 4). The normalized abundance of each identified allergen or homologue was retrieved from mass spectral data following normalization to the peptide DSRGNPTVEVELTTEKGVF (the most abundant identified peptide for yeast enolase included as a digestion control in all samples), which allowed comparison of relative abundances of proteins across runs. The gluten protein allergens were most abundant, followed by those belonging to the ATI family (Figure 4), which is consistent with the known protein composition of cereal seeds.^45^ The relative abundance of the gluten protein allergens did not map to the known relative abundances of the gluten proteins of α-gliadin > LMW-GS>γ-gliadin > HMW-GS > ω1,2-gliadins > ω5-gliadins.^46^

Thus, the most abundant allergen was the low-molecular-weight glutenin subunit allergen Tri a 36 of wheat, followed by the barley homologue of the HMW-GS allergen Tri a 26.0201 and the α-gliadin allergen Tri a 21 in wheat. The α-gliadin allergen Tri a 19, the γ-gliadin allergen Tri a 20 in wheat, and its homologues in rye and oats, together with the HMW-GS allergen Tri a 26.0201 in wheat and its homologues in rye and oats, had similar moderate levels of abundance. In contrast, the γ-hordein allergen Hor v 20 from barley and its homologues and the Tri a 20 homologue in barley were all of low abundance. Interestingly, homologues of allergens identified in the different cereal species were present at similar abundances to the allergens in the original species, apart from the Tri a 20 homologue in rye.

## Conclusions

Advances in the quantity and quality of genomic data available, for wheat and barley in particular, are supporting proteomic annotations, and the whole proteome analysis presented here provides protein-level evidence for proteins, including those from oats, which had only previously been imputed from genome or transcriptome data. However, although genome sequencing of wheat, barley, rye, and oats continues to advance, the quality and annotation of the assembly can limit the use of such data for proteomics analysis, and gaps still remain especially for crops, such as rye and oats, which limit the analysis that can be performed. Similarly, there are gaps in our knowledge of CTMs, since T-cell epitope mapping studies do not necessarily focus on celiacs with active disease or individuals with less common HLA types, such as DQ2.2 and DQ8. Indeed, many studies have focused on identifying CTMs in wheat, and it may be that there are novel CTMs still to be identified in other cereal species, such as barley and rye.^9^

Nevertheless, the proteomic profile of mature grain from wheat, barley, and rye clearly demonstrated the dominance of seed storage prolamins and allowed proteoforms to be identified, which confirmed the importance of gluten proteins as IgE-mediated food allergens. Putative cross-reactive homologues were also identified in barley, rye, and oats, confirming clinical observations of cross-reactive allergens between cereal species, especially wheat, barley, and rye. The ATIs, which are associated with the inhalant occupational allergy to flour known as Baker’s asthma, were of only moderate abundance, as were many minor wheat flour allergens. These findings suggest that if cereals, such as rye, barley, and oats, were more widely consumed in the future, their allergenic potential and potency could be revealed. This study also allowed direct comparison of the relative abundances of celiac-toxic motifs across different species and confirmed previous *in silico* analysis that the diversity and abundance of prolamins carrying CTMs present in wheat are consistent with its importance as a trigger for celiac disease, followed by barley, rye, and finally oats. In oats, together with the lack of potentially cross-reactive IgE allergens, this suggests that oats are more suitable for individuals with adverse reactions to wheat, supporting the decision of some regulators to exclude oats from the regulated list of cereals containing gluten. The more limited repertoire of proteins carrying CTMs also makes it more tractable to efforts to further reduce the levels by conventional breeding or the application of biotechnological approaches.

## Data Availability

The mass spectrometry proteomics data have been deposited to the ProteomeXchange Consortium via the PRIDE^47^ partner repository with the data set identifier PXD039539.

## Supporting Information Available

Supplementary Data File 1: proteins identified from mass spectral data within *T. aestivum*, *H. vulgare*, *S. cereal*, and *A. sativa* samples, using Progenesis QI for Proteomics and protein grouping (XLSX)
Supplementary Data File 2: peptides identified from mass spectral data within *T. aestivum*, *H. vulgare*, *S. cereal*, and *A. sativa* samples, using Progenesis QI for Proteomics and protein grouping (XLSX)
Supplementary Data File 3: proteins identified from mass spectral data within *T. aestivum*, *H. vulgare*, *S. cereal*, and *A. sativa* samples, using Progenesis QI for Proteomics and unique peptides only for protein identification (XLSX)
Supplementary Data File 4: peptides identified from mass spectral data within *T. aestivum*, *H. vulgare*, *S. cereal*, and *A. sativa* samples, using Progenesis QI for Proteomics and unique peptides only for protein identification (XLSX)
Supplementary Data File 5: principal component analysis loadings used to generate Figure 1 (XLSX)
Supplementary Data File 6: CD-active peptide identified in mass spectral data (XLSX)
Table S1: Ppincipal component analysis loadings for UniProt protein accessions identified using searching against Viridiplantae; Table S2: principal component analysis loadings for UniProt protein accessions identified using searching against Viridiplantae and tagged in the GluPro database of curated gluten protein sequences; Table S3: identification of cereal allergen homologues associated with IgE-mediated allergies using BLAST searching; Table S4: identification of cereal allergen homologues associated with IgE-mediated allergies using FASTA and a sliding 80mer window; Table S5: allergen isoforms identified from profiling cereals containing gluten; Figure S1: three-dimensional principal components analysis plots of protein abundance; Figure S2: gene ontology of identified proteins with aspect set to molecular function; Figure S3: digestion of cereal grain—quality control by 1D-PAGE; Figure S4: digestion of cereal grain—quality control by RP-HPLC; Figure S5: data processing pipeline for analysis of cereals containing gluten; Supplementary Figure S6: extracted ion chromatograms from two exemplar peptides identified in discovery mass spectrometry; Supporting Information S1: methods and quality control of chymotrypsin digestion (PDF)

## Abbreviations

ATI: α-amylase/trypsin inhibitors
BLAST: basic local alignment search tool
CD: celiac disease
BTEE: *N*-benzoyl-l-tyrosine ethyl ester
cDNA: complementary deoxyribonucleic acid
CM: chloroform/methanol
CTM: celiac-toxic motif
DIA: data-independent acquisition
DTT: dithiothreitol
FDA: Food and Drug Administration
FDR: false discovery rate
GO: gene ontology
HDMSe: high-definition mass spectrometry
HLA: human leukocyte antigen
HMW-GS: high molecular weight-glutenin subunit
HPLC: High-performance liquid chromatography
IgE: immunoglobulin E
LC-IM-MS: liquid chromatography–ion mobility-mass spectrometry
LC-MS: liquid chromatography–mass spectrometry
LDS,: lithium dodecyl sulfate
LMW-GS: low molecular weight-glutenin subunit
MES: 2-(*N*-morpholino)ethanesulfonic acid
mRNA: messenger ribonucleic acid
MS: mass spectrometry
PCA: principal component analysis
RP-HPLC: reversed-phase-high-performance liquid chromatography
UPLC: ultra-performance liquid chromatography
WDEIA: wheat-dependent exercise-induced anaphylaxis
WHO/IUIS: World Health Organization/International Union of Immunological Societies
