# Supplementary material for: Proteomic Profiling of Celiac-Toxic Motifs and Allergens in Cereals Containing Gluten
Source: J Proteome Res. 2025 Apr 15;24(5):2336–48. doi: 10.1021/acs.jproteome.3c00456 (PMC12053943; doi:10.1021/acs.jproteome.3c00456)
Supplement: Supplementary file 7 — pr3c00456_si_007.pdf [file pr3c00456_si_007.pdf]

Proteomic profiling of coeliac toxic motifs and allergens in cereals containing gluten

Matthew E. Daly[1], Xin Huang[2], Chiara Nitride[3], Christopher Hughes[4], Jaakko Tanskanen[5] Peter S. Shewry[6], Lee A. Gethings[1,4,7], E. N. Clare Mills[1,7]

[1] Manchester Institute of Biotechnology, School of Biological Sciences, Manchester Academic Health Sciences Centre, The University of Manchester, Princess Street, Manchester, M1 7DN, UK

[2] Department of Food and Nutrition, University of Helsinki, Agnes Sjöberginkatu 2, PL 66, FI-00014, Helsinki

[3] Department of Agricultural Sciences, University of Naples Federico II, Portici, 80055, Italy

[4] Waters Corporation, Stamford Avenue, Wilmslow, SK9 4AX, UK

[5] Natural Resources Institute (LUKE), Viikinkaari 1 00710 Helsinki, Finland

[6] Rothamsted Research, Harpenden, Herts, AL5 2JQ, UK.

[7] School of School of Biosciences and Medicine, The University of Surrey, Guildford, GU2 7XH, UK.

Supporting Information

Contents

Supporting Information S1 ....S3

Methods and quality control of chymotrypsin digestion..S3

Figure S1. Digestion of cereal grains quality control by 1D-PAGE...S5

Figure S2. RP-HPLC analysis of chymotryptic digests..S6

Supplementary Table S1. Principal component analysis loadings for UniProt protein accessions identified using searching against Viridiplantae..S8

Supplementary Table S2. Principal component analysis loadings for UniProt protein accessions identified using searching against Viridiplantae and tagged in the GluPro database of curated gluten protein sequences...S11

Supplementary Table S4. Identification of cereal allergen homologues associated with IgE-mediated allergies using FASTA and a sliding 80mer window...S19

Supplementary Table S5. Allergen isoforms identified from profiling cereals containing gluten. S24

Supplementary Figure S3. Three-dimensional principal components analysis plots of protein abundance S27

Supplementary Figure S4. Gene ontology of identified proteins with aspect set to molecular function. S28

Supplementary Figure S5. Data processing pipeline for analysis of cereals containing gluten using Progenesis QI for Proteomics S37

Supplementary Figure S6. Extracted ion chromatograms from two exemplar peptides identified in discovery mass spectrometry. S38

Supplementary Data file 1. Proteins identified from mass spectral data within T. aestivum, H. vulgare, S. cereal and A. sativa samples respectively, using Progenesis QI for Proteomics and protein grouping (XLSX). S39

Supplementary Data file 2. Peptides identified from mass spectral data within T. aestivum, H. vulgare, S. cereal and A. sativa samples respectively, using Progenesis QI for Proteomics and protein grouping (XLSX). S39

Supplementary Data file 3. Proteins identified from mass spectral data within T. aestivum, H. vulgare, S. cereal and A. sativa samples respectively, using Progenesis QI for Proteomics and unique peptides only for protein identification (XLSX). S39

Supplementary Data file 4. Peptides identified from mass spectral data within T. aestivum, H. vulgare, S. cereal and A. sativa samples respectively, using Progenesis QI for Proteomics and unique peptides only for protein identification (XLSX). S39

Supplementary Data file 5. Principal component analysis loadings used to generate Figure 1 (XLSX). S39

Supplementary Data file 6. CD-active peptide identified in mass spectral data (XLSX). S39

Supporting Information S1

Methods and quality control of chymotrypsin digestion.

Digestion quality control

Sample digestion was monitored by one-dimensional SDS polyacrylamide gel electrophoresis (1D-PAGE) and by reversed phase–high pressure liquid chromatography (RP-HPLC). For gel electrophoresis, both undigested (extract after reduction and alkylation) and digested samples were mixed 1:1 (v/v) with NuPAGE® LDS sample buffer containing 10mM DTT and heated at 90°C for 10 min. Twelve point five micrograms of protein were loaded per lane (4-12% NuPAGE® pre-cast Novex® gel) and the buffer chamber was filled with NuPAGE® MES buffer. Samples were separated for 35min at 200 V, 350 mA and 100W. Gels were fixed in 40% (v/v) methanol, 10% (w/v) trichloroacetic acid for 90min, washed 2 x 5min using 100mL distilled water prior to staining overnight with Simply Blue Safe Stain (Invitrogen, UK). After destaining using MilliQ water, the gel was subsequently imaged using a GE Healthcare Typhoon TRIO variable mode imager (GE Healthcare Lifesciences, Buckinghamshire, UK). The quality of the digestion workflow was assessed using 1D-PAGE (Supplementary Figure S3). This analysis demonstrated the degradation of intact proteins into polypeptides with a M_r < 2.5kDa, and also established the need for inclusion of RapiGest SF in the digestion buffer to ensure complete proteolysis of B hordeins present in barley. Digestion was also monitored using RP-HPLC on a Shimadzu Prominence UPLC system (Shimadzu, Kyoto, Japan). Reduced and alkylated sample extracts (control) and reduced, alkylated and digested sample extracts (digests) were diluted to 250µg/mL and filtered using Merck Millipore Millex™ Nonsterile Syringe Filters 0.45µm pore size (Fisher Scientific, Loughborough, UK). Fifty microliters of each cereal grain control and digest was separately loaded onto a Jupiter C18, 300Å, 5µm, 250 × 4.6mm column (Phenomenex, Macclesfield, UK). The column was equilibrated with 0.1% (v/v) formic acid in HPLC grade water (Buffer A), and a flow rate of

1mL/min was used throughout. Proteins were eluted using a gradient of 0.1% (v/v) formic acid in acetonitrile as buffer B. The elution gradient was as follows: 0 min 0% B, 0.5min 24% B, 20min 56% B, 20.1–24.1min 90% B, 24.2–30min 0% [31]. The absorbance of the eluate was monitored at 210nm. Chromatograms produced from RP-HPLC also demonstrated digestion of polypeptides as profiles were different between the control sample (no digestion) and digested sample (Supplementary Figure S4).

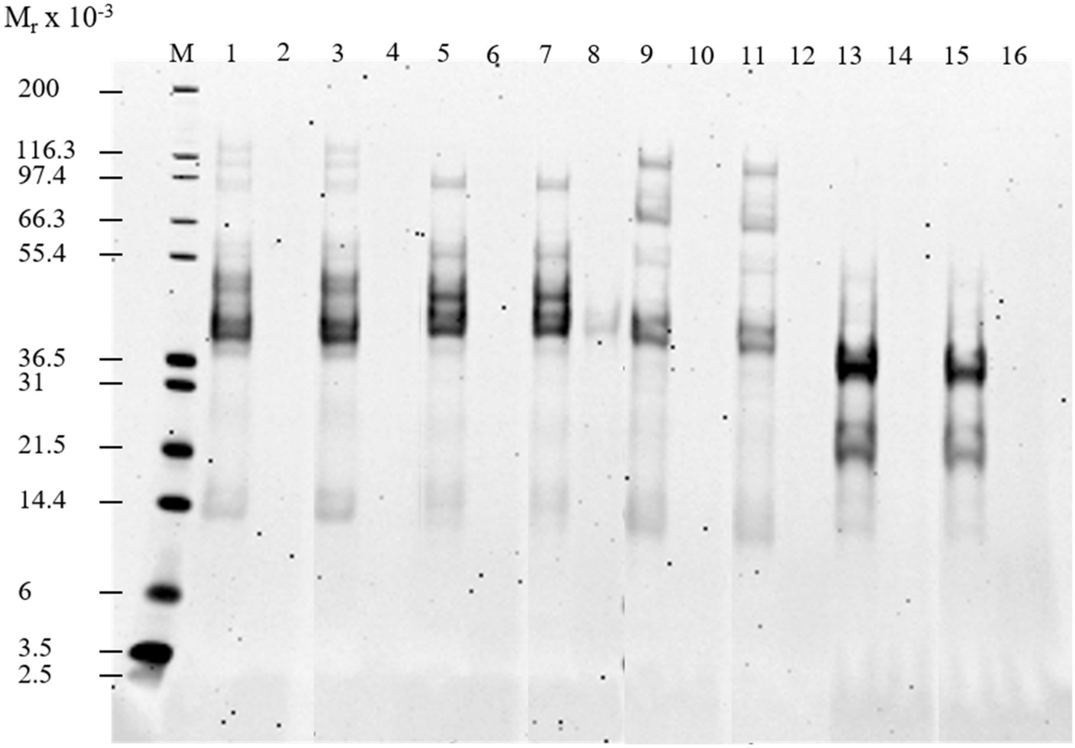

Figure S1. Digestion of cereal grains quality control by 1D-PAGE.

M = protein marker, lane 1, 5, 9 and 13 are controls of wheat, barley, rye and oats, respectively, that contained no chymotrypsin but did contain 0.1% (w/v) RapiGest SF. Lanes 2, 6, 10 and 14 are digested wheat, barley, rye and oats, respectively, containing 0.1% (w/v) RapiGest SF. Lanes 3, 7, 11 and 15 controls of wheat, barley, rye and oats, respectively, that contained no chymotrypsin and no RapiGest SF. Lanes 4, 8, 12 and 16 are digested wheat, barley, rye and oats, respectively, containing no RapiGest SF.

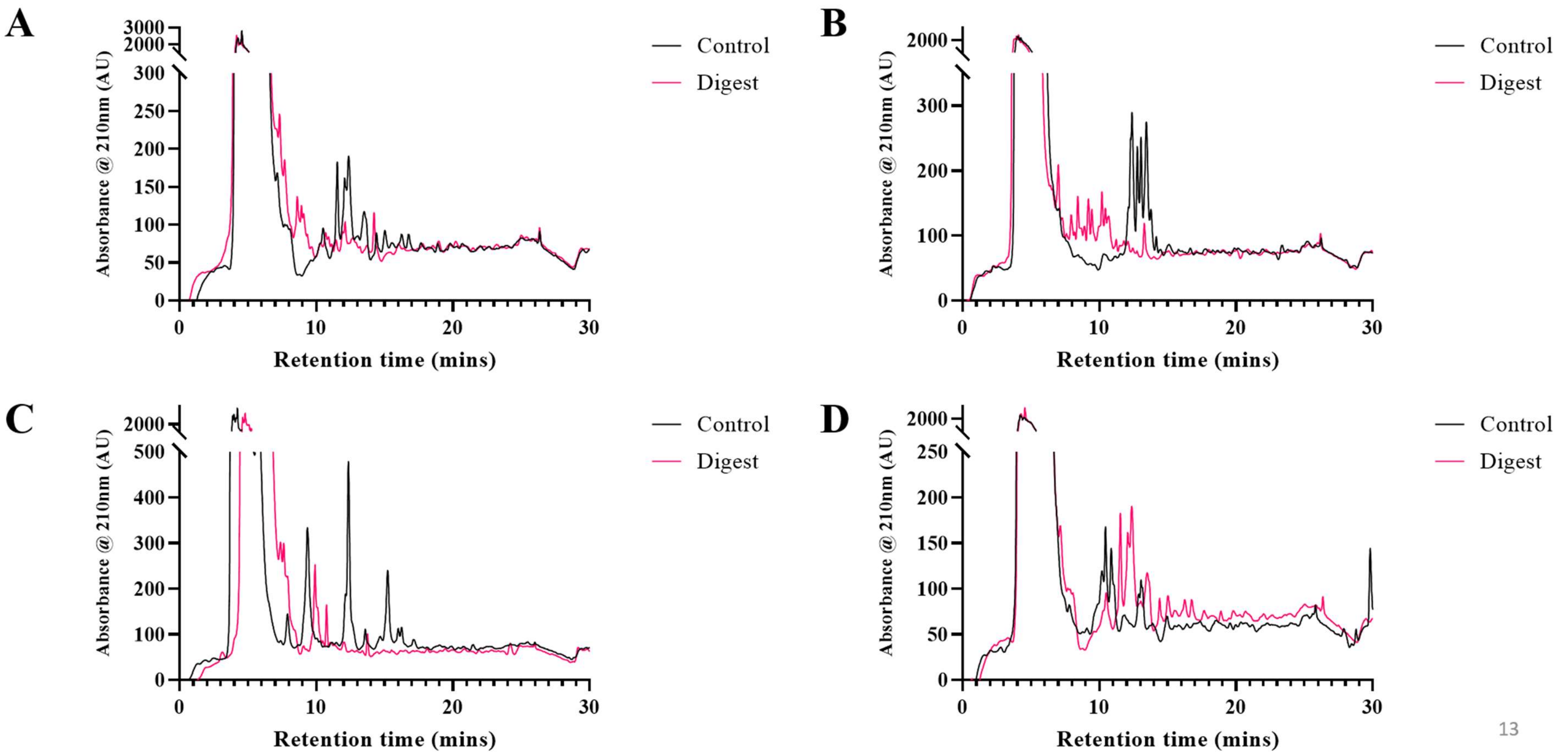

Figure S2. RP-HPLC analysis of chymotryptic digests.
chromatograms of a control sample (no chymotrypsin, black line) and a digested sample (pink line) of (A) *T. aestivum*, (B) *H. vulgare,* (C) *S. cereale* and (D) *A. sativa.*

Supplementary Table S1. Principal component analysis loadings for UniProt protein accessions identified using searching against Viridiplantae.

The protein accessions contributing to the separation observed on the PCA plot (Figure 1A, main paper) with loadings value, originating organism, protein description, protein score and the number of identified proteins. The pfam domain is included in parentheses if the protein description is uncharacterised and the domain information is available

PCA Loading	Accession	Loadings 1 value	Loadings 2 value	Loadings 3 value	Organism	Protein description (pfam domain)
**Largest +ve loadings 1 values**	A0A5B9Y471	0.070567	0.0065949	0.01773	*Triticum aestivum*	Gamma gliadin
	L7R599	0.06647	0.0095639	0.018819	*Triticum aestivum*	Gamma gliadin
	H8Y0P0	0.062451	-0.012653	0.032282	*Secale strictum ssp anatolicum*	Gamma prolamin
	A0A3L6SET2	0.057735	0.019362	0.022871	*Panicum milicaeum*	Globulin-1 S allele
	A0A0A9EFA0	0.054536	0.0085918	0.01608	*Arundo donax*	Uncharacterised protein (no pfam domain)
**Largest -ve loadings 1 values**	A0A1B2LQE6	-0.048429	-0.031911	-0.01388	*Avena magna*	Alpha amylase trypsin inhibitor
	A0A3B5ZP43	-0.039751	0.029039	0.007064	*Triticum aestivum*	Uncharacterised protein (Cupin_1)
	A0A287EP28	-0.037905	-0.014964	0.022358	*Hordeum vulgare ssp vulgare*	Uncharacterised protein (Obsolete entry)
	G1UH44	-0.035532	-0.0082732	-0.06415	*Hordeum vulgare ssp spontaneum*	Hordoindoline b-2

PCA Loading	Accession	Loadings 1 value	Loadings 2 value	Loadings 3 value	Organism	Protein description (pfam domain)
	A0A287EIM7	-0.034347	-0.041484	0.046014	*Hordeum vulgare ssp vulgare*	Uncharacterised protein (Obsolete entry)
**Largest +ve loadings 2 values**	A0A2T7C2V2	-0.0026511	0.054245	0.032242	*Panicum hallii var. hallii*	Uncharacterised protein (Cupin_1)
	A0A342D2K4	0.0013962	0.052724	0.032036	*Pogostemon stellatus*	DNA-directed RNA polymerase subunit beta
	A0A1U8B987	0.00048821	0.051505	0.031197	*Nelumbo nucifera*	Glyceraldehyde-3-phosphate dehydrogenase
	A0A3B5YPZ7	0.00022747	0.05062	0.030596	*Triticum aestivum*	AAI domain-containing protein
	B6UKP0	0.00067635	0.050131	0.030348	*Triticum aestivum*	Gamma gliadin
**Largest -ve loadings 2 values**	M0WPC3	-0.021245	-0.057387	0.043003	*Hordeum vulgare ssp vulgare*	Non-specific lipid-transfer protein
	A3RHE2	-0.018614	-0.056755	0.042553	*Hordeum vulgare ssp vulgare*	Deyhydrin
	A0A287HK37	0.015476	-0.055839	0.013547	*Hordeum vulgare ssp vulgare*	Sucrose synthase
	A0A2S3HJE7	-0.016086	-0.051531	0.038662	*Panicum hallii*	Uncharacterised protein (Oleosin)

PCA Loading	Accession	Loadings 1 value	Loadings 2 value	Loadings 3 value	Organism	Protein description (pfam domain)
	A0A5J9U427	-0.012352	-0.04741	0.035719	*Eragrostis curvula*	Uncharacterised protein
**Largest +ve loadings 3 values**	A0A2T7DAS7	-0.01898	0.020249	0.065754	*Panicum hallii var. hallii*	PPIase cyclophilin-type domain-containing protein
	A0A287TCE0	0.0163	0.020138	0.060531	*Hordeum vulgare ssp vulgare*	Peptidyl-prolyl cis-trans isomerase
	A0A0D3HJ84	-0.02518	-0.01273	0.05962	*Oryza barthii*	Protein disulfide-isomerase
	A0A4U6VJK4	-0.02249	-0.00257	0.057084	*Setaria viridis*	Phosphopyruvate hydratase
	P00924	0.002574	-0.00445	0.053415	*Saccharomyces cerevisiae*	Enolase 1
**Largest -ve loadings 3 values**	A0A446RJX9	-0.00253	-0.00244	-0.07866	*Triticum turgidum subsp. durum*	Sucrose synthase
	R4I506	-0.02375	-0.01427	-0.06951	*Avena sativa*	Vromindoline 1.3
	A0A0D9X204	0.000387	-0.00245	-0.06805	*Leersia perrieri*	Peroxiredoxin
	A0A0E0LND2	0.005382	-0.01652	-0.06468	*Oryza punctata*	Sucrose synthase
	G1UH44	-0.03553	-0.00827	-0.06415	*Hordeum vulgare subsp. spontaneum*	Hordoindoline b-2

Supplementary Table S2. Principal component analysis loadings for UniProt protein accessions identified using searching against Viridiplantae and tagged in the GluPro database of curated gluten protein sequences.

The protein accessions contributing to the separation observed on the PCA plot with loadings value, originating organism and GluPro classification (Daly, M., et al, 2020).

PCA Loading	Accession	Loadings 1 value	Loadings 2 value	Loadings 3 value	Organism	GluPro classification
**Largest +ve loadings 1 values**	Q0Q5D8	0.17016	0.011583	0.12576	*Triticum aestivum*	HMW-GS
	A0A023WHQ9	0.14952	-0.03352	-0.00448	*Triticum aestivum*	α-gliadin
	A0A446W0C7	0.13487	-0.03107	-0.00451	*Triticum turgidum* ssp *durum*	α-gliadin
	A0A446RKG6	0.13457	-0.03034	-0.00432	*Triticum turgidum* ssp *durum*	Avenin-like
	R9XVD4	0.12744	-0.03062	-0.00631	*Triticum aestivum*	LMW-GS
**Largest -ve loadings 1 values**	M0VH55	-0.08674	-0.17554	0.33482	*Hordeum vulgare* ssp *vulgare*	Avenin-like
	A0A287EFD4	-0.05412	-0.15957	0.005241	*Hordeum vulgare* ssp *vulgare*	B1 hordein
	I6TMV6	-0.05307	-0.15653	0.00515	*Hordeum vulgare* ssp *vulgare*	γ1 hordein

PCA Loading	Accession	Loadings 1 value	Loadings 2 value	Loadings 3 value	Organism	GluPro classification
	A0A287EFE1	-0.05256	-0.15481	0.005052	*Hordeum vulgare* ssp *vulgare*	B1 hordein
	I6SJ26	-0.05256	-0.15481	0.005054	*Hordeum vulgare* ssp *vulgare*	B3 hordein
**Largest +ve loadings 2 values**	H8Y0K4	-0.01431	0.35324	0.30227	*Secale cereale*	γ 40k secalin
	D0IQ07	-0.02613	0.088388	0.20913	*Triticum aestivum*	LMW-GS
	A0A3B6TIG9	-0.026	0.087948	0.20797	*Triticum aestivum*	Avenin-like
	F2EGD5	-0.02595	0.087776	0.20742	*Hordeum vulgare* ssp *vulgare*	Avenin-like
	Q94IL3	-0.02592	0.087673	0.20735	*Secale cereale*	HMW secalin subunit
**Largest -ve loadings 2 values**	M0VH55	-0.08674	-0.17554	0.33482	*Hordeum vulgare* ssp *vulgare*	Avenin-like
	A0A287EFD4	-0.05412	-0.15957	0.005241	*Hordeum vulgare* ssp *vulgare*	B1 hordein
	I6TMV6	-0.05307	-0.15653	0.00515	*Hordeum vulgare* ssp *vulgare*	γ1 hordein
	A0A287EFE1	-0.05256	-0.15481	0.005052	*Hordeum vulgare* ssp *vulgare*	B1 hordein
	I6SJ26	-0.05256	-0.15481	0.005054	*Hordeum vulgare* ssp *vulgare*	B3 hordein

PCA Loading	Accession	Loadings 1 value	Loadings 2 value	Loadings 3 value	Organism	GluPro classification
**Largest +ve loadings 3 values**	M0VH55	-0.08674	-0.17554	0.33482	*Hordeum vulgare* ssp *vulgare*	Avenin-like
	H8Y0K4	-0.01431	0.35324	0.30227	*Secale cereale*	γ 40k secalin
	D3XQB8	0.061236	0.078479	0.23325	*Secale cereale*	HMW secalin subunit
	D0IQ07	-0.02613	0.088388	0.20913	*Triticum aestivum*	HMW-GS
	A0A3B6TIG9	-0.026	0.087948	0.20797	*Triticum aestivum*	Avenin-like
**Largest -ve loadings 3 values**	L0L5I0	-0.02988	0.086811	-0.21169	*Avena sativa*	B type avenin
	I4EP88	-0.02983	0.086692	-0.21125	*Avena sativa*	B type avenin
	L0L833	-0.02965	0.086183	-0.20996	*Avena sativa*	A type avenin
	L0L6K1	-0.0295	0.085701	-0.20882	*Avena sativa*	C type avenin
	L0L5H3	-0.02938	0.085335	-0.20753	*Avena sativa*	A type avenin

Supplementary Table S3. Identification of cereal allergen homologues associated with IgE-mediated allergies using BLAST searching.

Cereal allergens were retrieved form the WHO/IUIS allergen nomenclature database and homologues in other cereal grains identified using full sequence BLAST searching of the whole proteome identified using Viridiplantae. The value in brackets indicate the sequence identity. No allergens are attributed to oats in the WHO/IUIS allergen nomenclature database.

Organism	Protein type	WHO/IUIS allergen designation	Named UniProt accession	Homologue			
				**Wheat (*Triticum aestivum*)**	**Barley (*Hordeum vulgare*)**	**Rye (*Secale cereale*)**	**Oats (*Avena sativa*)**
Wheat (*Triticum aestivum*)	Profilin	Tri a 12	P49234		F2E5Q1 (93.1%)		
			B6EF35		F2E5Q1 (94.7%)		
			P49233		F2E5Q1 (93.9%)		
			P49232		F2E5Q1 (93.9%)		
	Non-specific lipid transfer protein 1	Tri a 14	D2T2K2		F2CY84 (88%)	Q155V1 (41.3%)	
	Monomeric alpha-amylase inhibitor 0.28	Tri a 15	D2TGC3		P13691 (63.6%)	C3VWW2 (59.2%)	
	Beta-amylase	Tri a 17	P93594		Q4VM11 (97.6)	Q08335 (96.6%)	
	Agglutinin isolectin 1	Tri a 18	P10968		P15312 (92.9%)	Q9FRV1 (45.1%	Q38769 (31.5%)

Organism	Protein type	WHO/IUIS allergen designation	Named UniProt accession	Homologue			
				Wheat (*Triticum aestivum*)	Barley (*Hordeum vulgare*)	Rye (*Secale cereale*)	Oats (*Avena sativa*)
	Omega-5 gliadin	Tri a 19	Q402I5		Q41210 (47.9%)	K7WJK0 (52.2%)	
	Gamma gliadin	Tri a 20	Q9SYX8		A0A8I6WNF0 (50.8%)	H8Y0P6 (79.3%)	Q09114 (45.8%)
			A0A060N479		A0A8I6WNF0 (50.8%)	H8Y0P6 (79.3%)	Q09114 (45.8%)
	Alpha/beta gliadin	Tri a 21	D2T2K3				
	Thioredoxin	Tri a 25	Q9LDX4		Q7XZK2 (89.9%)	A0A1C6ZYA9 (46.2%)	
	High molecular weight glutenin subunit	Tri a 26	Q45R38		Q84LE9 (40.6%)	Q93WF0 (63.1%)	I4EP64 (41%)
			P10388		Q84LE9 (48.5%)	Q94IL5 (58.9%)	I4EP64 (47.2%)
	Thiol reductase homologue	Tri a 27	Q7Y1Z2		A0A8I7BCH5 (87.7%)	A0A1C9T8F8 (34.1%)	
	Dimeric alpha amylase inhibitor 0.19	Tri a 28	Q4W0V7		C3VX00 (100%)	C3VWW4 (100%)	
	Tetrameric alpha-amylase inhibitor CM1/CM2	Tri a 29	D2TGC2		P28041 (81.7%)	Q45FA6 (47.8%)	A0A1B2LQC0 (56.3%)

Organism	Protein type	WHO/IUIS allergen designation	Named UniProt accession	Homologue			
				**Wheat (*Triticum aestivum*)**	**Barley (*Hordeum vulgare*)**	**Rye (*Secale cereale*)**	**Oats (*Avena sativa*)**
			C7C4X0		P28041 (84.2%)	Q45FA6 (52.8%)	A0A1B2LQC9 (56.2%)
	Tetrameric alpha amylase inhibitor CM3	Tri a 30	P17314		P11643 (83%)	Q45FA6 (45.8%)	A0A1B2LQD9 (50.9%)
	Triosephosphate-isomerase	Tri a 31	Q9FS79		F2EHF8 (94.9%)	P46226 (96.4%)	
	1-cys-peroxiredoxin	Tri a 32	Q6W8Q2		P52572 (98.2%)		
	Serpin	Tri a 33	Q9ST57		F2DHX6 (73.6%)	A0A3G1MTQ3 (31.7%)	A0A0U3DJ23 (31.7%)
	Glyceraldehyde-3-phosphate-dehydrogenase	Tri a 34	C7C4X1		P26517 (99.7%)	I3RXT5 (89.5%)	A0A411EWM9 (87%)
	Dehydrin	Tri a 35	D2TE72		T1TDJ6 (52.4%)	F1DB88 (51.3%)	
	Low molecular weight glutenin subunit	Tri a 36	B2Y2Q7		I6TEV5 (57.6%)	E5KZQ2 (43.32%)	
	Alpha purothionin	Tri a 37	Q9T0P1		F2EE63 (84.6%)	Q9ZNY5 (86.8%)	Q8LT03 (54.7%)
	Serine protease inhibitor-like protein	Tri a 39	J7QW61		A8V3X4 (86.9%)		

Organism	Protein type	WHO/IUIS allergen designation	Named UniProt accession	Homologue			
				**Wheat (*Triticum aestivum*)**	**Barley (*Hordeum vulgare*)**	**Rye (*Secale cereale*)**	**Oats (*Avena sativa*)**
	Chloroform/methanol-soluble (CM) 17 protein [alpha-amylase inhibitor]	Tri a 40	Q41540		P32936 (82.5%)	Q45FA6 (88%)	A0A1B2LQD6 (55.9%)
	Mitochondrial ubiquitin ligase activator of NFKB 1	Tri a 41	A0A0G3F2P1		F2EFJ6 (98.3%)	A0A2U9K6M7 (46.2%)	
	Hypothetical protein from cDNA	Tri a 42	A0A0G3F2F5		M0XDM5 (90.5%)	A0A1C9T8W1 (52.9%)	
	Hypothetical protein from cDNA	Tri a 43	A0A0G3F5F7		A0A8I7BEI9 (94.4%)		
	Endosperm transfer cell specific PR60 precursor	Tri a 44	A0A0G3F720		F2EEH7 (86.9%)		
	Elongation factor 1 (EIF1)	Tri a 45	A0A0G3F715		A0A8I6WYJ6 (95.5%)	A0A0S2LJ14 (34.6%)	
Barley (*Hordeum vulgare*)	No description	Hor v 5	O04828	A0A3B6U382 (88.3%)		F4MJM3 (71.9%)	I4IY75 (65.6%)
	Profilin	Hor v 12	P52184	E2GJB9 (96.9%)		A0A2U9N6P3 (34.2%)	
	Alpha-amylase inhibitor BMAI-1 precursor	Hor v 15	P16968	A0A3B6PE52 (52.8%)		C3VWV8 (39.7%)	A0A1B2LQA9 (38.9%)
	Alpha-amylase	Hor v 16					

Organism	Protein type	WHO/IUIS allergen designation	Named UniProt accession	Homologue			
				**Wheat (*Triticum aestivum*)**	**Barley (*Hordeum vulgare*)**	**Rye (*Secale cereale*)**	**Oats (*Avena sativa*)**
	Beta-amylase	Hor v 17	No isoform listed				
	Gamma-hordein 3	Hor v 20	P80198	A0A2U8JD37 (71.2%)		K7WF86 (40.6%)	I4EP78 (44.4%)
Rye (*Secale cereale*)	Group 5 grass pollen allergen	Sec c 5	F4MJM3	A0A3B6U9A9 (86.1%)	F2EL92 (78.4%)		I4IY75 (65.1%)
	Gamma-secalin	Sec c 20	Q9S8B0	P21292 (95.7%)			L0L6J7 (58.3%)
			Q9S8A7	L7R918 (79.3%)	P80198 (61.9%)		
	Dimeric alpha-amylase/trypsin inhibitor	Sec c 38	Q9S8H2	A0A1D5UB33 (52.4%)	A0A8I6XS48 (78.3%)		

Supplementary Table S4. Identification of cereal allergen homologues associated with IgE-mediated allergies using FASTA and a sliding 80mer window.

Cereal allergens were retrieved form the WHO/IUIS allergen nomenclature database and homologues in other cereal grains identified using a sliding 80mer window and FASTA searching of the whole proteome identified using Viridiplantae. Values inside the brackets indicate the best % identity hit and the number of hits in total with >35% sequence identity. Blue shaded cells denoted isoforms also identified using BLAST searching (see Supplementary Table S3)

Organism	Protein type	WHO/IUIS allergen designation	Named UniProt accession	Homologue			
				**Wheat (*Triticum aestivum*)**	**Barley (*Hordeum vulgare*)**	**Rye (*Secale cereale*)**	**Oats (*Avena sativa*)**
Wheat (*Triticum aestivum*)	Profilin	Tri a 12	P49234		F2E5Q1 (97.5%, 51)		
			B6EF35		F2E5Q1 (97.5%, 51)		A0A1B2LQC9 (66.2%, 40)
			P49233		F2E5Q1 (97.5%, 51)		
			P49232		F2E5Q1 (97.5%, 51)		
	Non-specific lipid transfer protein 1	Tri a 14	D2T2K2		F2CY84 (91.2%, 12)	Q155V1 (40%, 12)	
	Monomeric alpha-amylase inhibitor 0.28	Tri a 15	D2TGC3		F2EI31 (68.4%, 41)	C3VWW2 (65.4%, 41)	A0A1B2LQD6 (42.6%, 29)
	Beta-amylase	Tri a 17	P93594		O23978 (100%, 423)	Q08335 (100%, 423)	
	Agglutinin isolectin 1	Tri a 18	P10968		A0A8I6WXU4 (95%, 132)	Q9AXR9 (72.2%, 132)	
	Omega-5 gliadin	Tri a 19	Q402I5				F4MJY1 (56.8%, 351)

Organism	Protein type	WHO/IUIS allergen designation	Named UniProt accession	Homologue			
				Wheat (*Triticum aestivum*)	Barley (*Hordeum vulgare*)	Rye (*Secale cereale*)	Oats (*Avena sativa*)
	Gamma gliadin	Tri a 20	Q9SYX8		Q40053 (72.9%, 131)	H8Y0P1 (86.3%, 218)	Q09072 (65.4%, 165)
			A0A060N479		Q40053 (72.9%, 112)	H8Y0P1 (86.3%, 199)	Q09072 (65.4%, 164)
	Alpha/beta gliadin	Tri a 21	D2T2K3			I3RXX8 (95%, 201)	F2Q9W4 (63.6%, 172)
	Thioredoxin	Tri a 25	Q9LDX4		F2DV81 (96.3%, 45)	A0A1C6ZYA9 (49.2%, 45)	
	High molecular weight glutenin subunit	Tri a 26	Q45R38			Q94IK6 (92.3%, 715)	L0L6K1 (45.2%, 54)
			P10388			D3XQB7 (91.1%, 768)	F4MJY2 (50%, 130)
	Thiol reductase homologue	Tri a 27	Q7Y1Z2		A0A8I7BCH5 (96.3%, 123)		
	Dimeric alpha amylase inhibitor 0.19	Tri a 28	Q4W0V7		C3VX00 (100%, 39)	C3VWW4 (100%, 39)	A0A1B2LQC9 (66.7%, 36)
	Tetrameric alpha-amylase inhibitor CM1/CM2	Tri a 29	D2TGC2		A0A8I6YCK0 (82.5%, 40)		A0A1B2LQC9 (62.3%, 40)
			C7C4X0		A0A8I6YCK0 (85%, 40)		A0A1B2LQC9 (66.2%, 40)

Organism	Protein type	WHO/IUIS allergen designation	Named UniProt accession	Homologue			
				**Wheat** (***Triticum aestivum***)	**Barley** (***Hordeum vulgare***)	**Rye** (***Secale cereale***)	**Oats** (***Avena sativa***)
	Tetrameric alpha amylase inhibitor CM3	Tri a 30	P17314		A0A8I6YKH0 (91.2%, 88)	C3VWW2 (70.6%, 78)	A0A1B2LQD6 (63.6%, 88)
	Triosephosphate-isomerase	Tri a 31	Q9FS79		F2EHF8 (97.5%, 173)	P46226 (100%, 173)	
	1-cys-peroxiredoxin	Tri a 32	Q6W8Q2		A0A8I6WHE5 (100%, 138)	O81480 (46.8%, 50)	
	Serpin	Tri a 33	Q9ST57				
	Glyceraldehyde-3-phosphate-dehydrogenase	Tri a 34	C7C4X1		A0A067YHY9 (100%, 105)	B3FIA4 (100%, 82)	A0A411EWM9 (95%, 246)
	Dehydrin	Tri a 35	D2TE72		A0A8I6YE80 (60.9%, 13)	F1DB88 (53.8%, 32)	
	Low molecular weight glutenin subunit	Tri a 36	B2Y2Q7			E5KZQ5 (65.8%, 283)	
	Alpha purothionin	Tri a 37	Q9T0P1		P01545 (86.7%, 57)	Q9ZNY5 (87.3%, 57)	Q8LT02 (61.3%, 57)
	Serine protease inhibitor-like protein	Tri a 39	J7QW61		P08626 (89.6%, 4)		
	Chloroform/methanol-soluble (CM) 17 protein [alpha-amylase inhibitor]	Tri a 40	Q41540		P32936 (89.7%, 63)	Q45FA6 (94.9%, 63)	A0A1B2LQD6 (67.9%, 63)

Organism	Protein type	WHO/IUIS allergen designation	Named UniProt accession	Homologue			
				Wheat (*Triticum aestivum*)	Barley (*Hordeum vulgare*)	Rye (*Secale cereale*)	Oats (*Avena sativa*)
	Mitochondrial ubiquitin ligase activator of NFKB 1	Tri a 41	A0A0G3F2P1				
	Hypothetical protein from cDNA	Tri a 42	A0A0G3F2F5				
	Hypothetical protein from cDNA	Tri a 43	A0A0G3F5F7		A0A8I7BEI9 (95%, 28)		
	Endosperm transfer cell specific PR60 precursor	Tri a 44	A0A0G3F720				
	Elongation factor 1 (EIF1)	Tri a 45	A0A0G3F715		A0A8I6WYJ6 (96.3%, 9)		
Barley (*Hordeum vulgare*)	No description	Hor v 5	O04828	A0A3B6NJH4 (100%, 194)		F4MJM3 (79.2%, 188)	
	Profilin	Hor v 12	P52184	A0A3B6TKM4 (97.5%, 51)			
	Alpha-amylase inhibitor BMAI-1 precursor	Hor v 15	P16968	I6PWK7 (58.8%, 66)			A0A1B2LQC9 (41.4%, 25)
	Alpha-amylase	Hor v 16	No isoform listed				
	Beta-amylase	Hor v 17					
	Gamma-hordein 3	Hor v 20	P80198	I7KM78 (80%, 209)		H8Y0P6 (67.1%, 177)	F2Q9W4 (59.7%, 139)

Organism	Protein type	WHO/IUIS allergen designation	Named UniProt accession	Homologue			
				**Wheat** (***Triticum aestivum***)	**Barley** (***Hordeum vulgare***)	**Rye** (***Secale cereale***)	**Oats** (***Avena sativa***)
Rye (*Secale cereale*)	Group 5 grass pollen allergen	Sec c 5	F4MJM3	A0A3B6PHA5 (93.8%, 212)	A0A8I6XR69 (83.8%, 212)		I4IY75 (76.2%, 212)
	Gamma-secalin	Sec c 20	Q9S8B0				
			Q9S8A7				
	Dimeric alpha-amylase/trypsin inhibitor	Sec c 38	Q9S8H2				

Supplementary Table S5. Allergen isoforms identified from profiling cereals containing gluten.

Mass spectral libraries were searched and allergen isoforms designated in the WHO/IUIS allergen nomenclature database or homologues identified through BLAST or 80mer/FASTA searching were identified. Boxes shaded in green indicate that accession was identified in mass spectral data using either protein grouping or proteins with at least one unique peptide, placed in separate columns. The confidence score of the identified protein accession is shown within parentheses after the accession name.

**Organism**	**WHO/IUIS allergen designation**	**Named UniProt accession**		**Homologue**															
				**Wheat (*Triticum aestivum*)**				**Barley (*Hordeum vulgare*)**				**Rye (*Secale cereale*)**				**Oats (*Avena sativa*)**			
				**BLAST**		**80mer/FASTA**		**BLAST**		**80mer/FASTA**		**BLAST**		**80mer/FASTA**		**BLAST**		**80mer/FASTA**	
		**Protein grouping**	**Unique peptide**	**Protein grouping**	**Unique peptide**	**Protein grouping**	**Unique peptide**	**Protein grouping**	**Unique peptide**	**Protein grouping**	**Unique peptide**	**Protein grouping**	**Unique peptide**	**Protein grouping**	**Unique peptide**	**Protein grouping**	**Unique peptide**	**Protein grouping**	**Unique peptide**
Wheat (*Triticum aestivum*)	Tri a 12	P49234	P49234					F2E5Q1	F2E5Q1	F2E5Q1	F2E5Q1								
		B6EF35	B6EF35					F2E5Q1	F2E5Q1	F2E5Q1	F2E5Q1			Q7M218 (29.7)	Q7M218 (29.7)			A0A1B2LQC9	A0A1B2LQC9 (125.8)
		P49233	P49233					F2E5Q1	F2E5Q1	F2E5Q1	F2E5Q1								
		P49232	P49232					F2E5Q1	F2E5Q1	F2E5Q1	F2E5Q1								
	Tri a 14	D2T2K2	D2T2K2					F2CY84	F2CY84	F2CY84	F2CY84	Q155V1	Q155V1	Q155V1	Q155V1	Q9S8W4	Q9S8W4	Q9S8W4	Q9S8W4
	Tri a 15	D2TGC3	D2TGC3					P13691	P13691 (160.3)	F2EI31 (161.8)	F2EI31	C3VWW2	C3VWW2	C3VWW2	C3VWW2			A0A1B2LQD6 (161.0)	A0A1B2LQD6 (161.0)
	Tri a 17	P93594	P93594					Q4VM11	Q4VM11	O23978	O23978	Q08335	Q08335	Q08335	Q08335				
	Tri a 18	P10968	P10968					P15312	P15312	A0A8I6WXU4	A0A8I6WXU4	Q9FRV1 (133.4)	Q9FRV1 (129.4)	Q9AXR9	Q9AXR9	Q38769 (81.7)	Q38769 (81.7)		
	Tri a 19	Q402I5	Q402I5					Q41210 (324.8)	Q41210 (324.8)	Q7M1Z4	Q7M1Z4	K7WJK0	K7WJK0	Q9S8B0	Q9S8B0			F4MJY1	F4MJY1

	Tri a 20	Q9SY X8	Q9SY X8					A0A8I6 WNF0	A0A8I6 WNF0	Q40053 (293.5)	Q40053 (293.5)	H8Y0P6 (52.1)	H8Y0P6 (52.1)	H8Y0P1	H8Y0P1	Q09114 (247.5)	Q09114 (247.5)	Q09072	Q09072
		A0A0 60N47 9 (267.7)	A0A0 60N47 9 (249.1)					A0A8I6 WNF0	A0A8I6 WNF0	Q40053 (293.5)	Q40053 (293.5)	H8Y0P6 (52.1)	H8Y0P6 (52.1)	H8Y0P1	H8Y0P1	Q09114 (247.5)	Q09114 (247.5)	Q09072	Q09072
	Tri a 21	D2T2 K3 (322.6)	D2T2 K3 (297.2)							P82936	P82936			I3RXX8	I3RXX8			F2Q9W4	F2Q9W4
	Tri a 25	Q9LD X4 (19.7)	Q9LD X4 (19.7)					Q7XZK 2	Q7XZK 2 (6.8)	F2DV81 (6.8)	F2DV81 (6.3)	A0A1C6 ZYA9	A0A1C6 ZYA9	A0A1C6 ZYA9	A0A1C6 ZYA9				
	Tri a 26	Q45R 38 (484.6)	Q45R 38					Q84LE9 (542.9)	Q84LE9 (542.9)	G9IHV7	G9IHV7	Q93WF0	Q93WF0	Q94IK6	Q94IK6	I4EP64	I4EP64	L0L6K1 (214.7)	L0L6K1 (214.7)
		P1038 8	P1038 8					Q84LE9 (542.9)	Q84LE9 (542.9)	G9IHV7	G9IHV7	Q94IL5 (357.6)	Q94IL5 (357.6)	D3XQB 7	D3XQB 7	I4EP64	I4EP64	F4MJY2	F4MJY2
	Tri a 27	Q7Y1 Z2 (61.3)	Q7Y1 Z2 (61.3)					A0A8I7 BCH5	A0A8I7 BCH5	A0A8I7 BCH5	A0A8I7 BCH5	A0A1C9 T8F8	A0A1C9 T8F8						
	Tri a 28	Q4W0 V7	Q4W0 V7					C3VX00	C3VX00	C3VX00	C3VX00	C3VWW 4	C3VWW 4	C3VWW 4	C3VWW 4			A0A1B2 LQC9	A0A1B2 LQC9 (125.8)
	Tri a 29	D2TG C2 (81.8)	D2TG C2 (81.8)					P28041 (145.7)	P28041 (145.7)	A0A8I6 YCK0	A0A8I6 YCK0	Q45FA6 (147.2)	Q45FA6 (144.6)	Q7M218 (29.7)	Q7M218 (29.7)	A0A1B2 LQC0	A0A1B2 LQC0 (84.9)	A0A1B2 LQC9	A0A1B2 LQC9 (125.8)
		C7C4 X0	C7C4 X0					P28041 (145.7)	P28041 (145.7)	A0A8I6 YCK0	A0A8I6 YCK0	Q45FA6 (147.2)	Q45FA6 (144.6)	Q7M218 (29.7)	Q7M218 (29.7)	A0A1B2 LQC9	A0A1B2 LQC9 (125.8)	A0A1B2 LQC9	A0A1B2 LQC9 (125.8)
	Tri a 30	P1731 4	P1731 4 (148.5)					P11643 (171.4)	P11643 (171.4)	A0A8I6 YKH0	A0A8I6 YKH0	Q45FA6 (147.2)	Q45FA6 (144.6)	C3VWW 2	C3VWW 2	A0A1B2 LQD9 (126.5)	A0A1B2 LQD9	A0A1B2 LQD6 (161.0)	A0A1B2 LQD6 (161.0)
	Tri a 31	Q9FS7 9	Q9FS7 9					F2EHF8 (107.5)	F2EHF8 (107.5)	F2EHF8 (107.5)	F2EHF8 (106.2)	P46226 (71.3)	P46226 (71.3)	P46226 (71.3)	P46226 (71.3)				
	Tri a 32	Q6W8 Q2 (53.2)	Q6W8 Q2					P52572 (182.6)	P52572 (182.6)	A0A8I6 WHE5	A0A8I6 WHE5			O81480	O81480				
	Tri a 33	Q9ST 57 (147.4)	Q9ST 57 (147.4)					F2DHX 6	F2DHX 6	Q7M273	Q7M273	A0A3G1 MTQ3	A0A3G1 MTQ3			A0A0U3 DJ23	A0A0U3 DJ23		
	Tri a 34	C7C4 X1 (142.2)	C7C4 X1					P26517	P26517	A0A067 YHY9	A0A067 YHY9	I3RXT5	I3RXT5	B3FIA4	B3FIA4	A0A411 EWM9	A0A411 EWM9	A0A411 EWM9	A0A411 EWM9
	Tri a 35	D2TE 72	D2TE 72					T1TDJ6	T1TDJ6	A0A8I6 YE80	A0A8I6 YE80	F1DB88	F1DB88 (13.0)	F1DB88	F1DB88				

	Tri a 36	B2Y2Q7 (466.5)	B2Y2Q7					I6TEV5	I6TEV5	Q9SAT9	Q9SAT9	E5KZQ2	E5KZQ2	E5KZQ5	E5KZQ5			Q09097	Q09097
	Tri a 37	Q9T0P1	Q9T0P1 (65.3)					F2EE63	F2EE63	P01545 (143.6)	P01545 (139.1)	Q9ZNY5 (48.8)	Q9ZNY5 (48.8)	Q9ZNY5 (48.8)	Q9ZNY5 (48.8)	Q8LT03	Q8LT03	Q8LT02	Q8LT02
	Tri a 39	J7QW61	J7QW61					A8V3X4	A8V3X4	P08626	P08626								
	Tri a 40	Q41540	Q41540					P32936	P32936 (134.5)	P32936	P32936 (134.5)	Q45FA6 (147.2)	Q45FA6 (144.6)	Q45FA6 (147.2)	Q45FA6	A0A1B2LQD6 (161.0)	A0A1B2LQD6 (161.0)	A0A1B2LQD6 (161.0)	A0A1B2LQD6 (161.0)
	Tri a 41	A0A0G3F2P1	A0A0G3F2P1					F2EFJ6	F2EFJ6			A0A2U9K6M7	A0A2U9K6M7						
	Tri a 42	A0A0G3F2F5	A0A0G3F2F5					M0XDM5	M0XDM5			A0A1C9T8W1	A0A1C9T8W1						
	Tri a 43	A0A0G3F5F7	A0A0G3F5F7					A0A8I7BEI9	A0A8I7BEI9	A0A8I7BEI9	A0A8I7BEI9								
	Tri a 44	A0A0G3F720	A0A0G3F720					F2EEH7 (90.8)	F2EEH7 (90.8)	F2EKS0	F2EKS0								
	Tri a 45	A0A0G3F715	A0A0G3F715					A0A8I6WYJ6	A0A8I6WYJ6	A0A8I6WYJ6	A0A8I6WYJ6	A0A0S2LJ14	A0A0S2LJ14						
Barley (*Hordeum vulgare*)	Hor v 5	O04828	O04828	A0A3B6U382	A0A3B6U382	A0A3B6NJH4	A0A3B6NJH4					F4MJM3	F4MJM3	F4MJM3	F4MJM3	I4IY75	I4IY75		
	Hor v 12	P52184	P52184	E2GJB9	E2GJB9	A0A3B6TKM4	A0A3B6TKM4					A0A2U9N6P3	A0A2U9N6P3						
	Hor v 15	P16968 (48.2)	P16968 (48.9)	A0A3B6PE52 (48.9)	A0A3B6PE52 (48.9)	I6PWK7 (46.9)	I6PWK7 (46.9)					C3VWV8 (178.4)	C3VWV8 (164.4)	Q7M221	Q7M221	A0A1B2LQA9 (115.3)	A0A1B2LQA9 (115.3)	A0A1B2LQC9	A0A1B2LQC9 (125.8)
	Hor v 20	P80198	P80198 (176.3)	A0A2U8JD37 (116.1)	A0A2U8JD37 (116.1)	I7KM78 (134.1)	I7KM78					K7WF86	K7WF86	H8Y0P6 (52.1)	H8Y0P6 (52.1)	I4EP78 (125.0)	I4EP78	F2Q9W4	F2Q9W4
Rye (*Secale cereale*)	Sec c 5	F4MJM3	F4MJM3	A0A3B6U9A9	A0A3B6U9A9	A0A3B6PHA5	A0A3B6PHA5	F2EL92	F2EL92	A0A8I6XR69	A0A8I6XR69					I4IY75	I4IY75	I4IY75	I4IY75
	Sec c 20	Q9S8B0	Q9S8B0	P21292	P21292 (159.5)			Q7M1Z5	Q7M1Z5							L0L6J7	L0L6J7		
		Q9S8A7	Q9S8A7	L7R918	L7R918			P80198	P80198 (176.3)										
	Sec c 38	Q9S8H2	Q9S8H2	A0A1D5UB33 (53.0)	A0A1D5UB33 (53.0)			A0A8I6XS48	A0A8I6XS48										

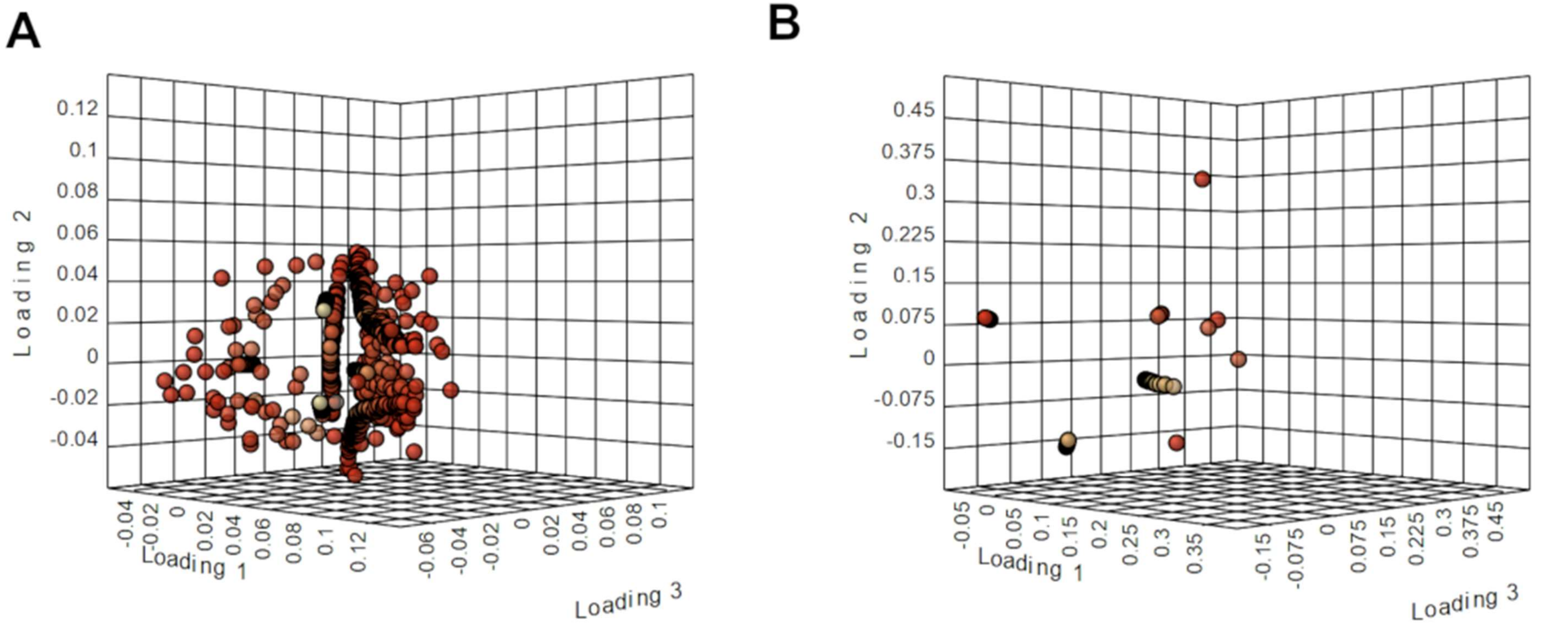

Supplementary Figure S3. Three-dimensional principal components analysis plots of protein abundance
(A) 3D PCA loadings plot of protein abundance from searching against in Viridiplantae. (B) 3D PCA loadings plot of protein abundance from those proteins present in GluPro v 6.1.

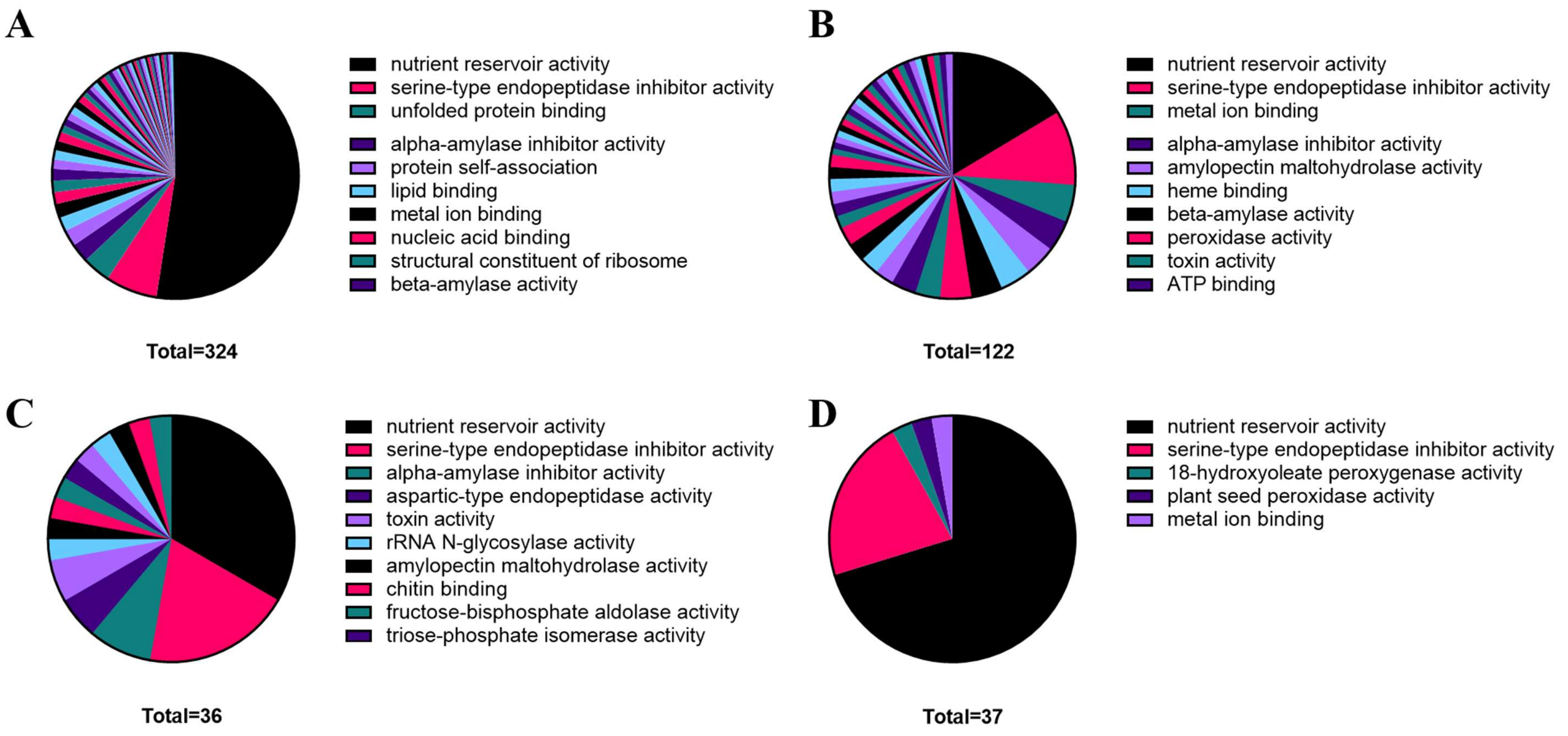

Supplementary Figure S4. Gene ontology of identified proteins with aspect set to molecular function.
The first ten most numerous GO terms for each grain is listed in the legend (A) *T. aestivum*, (B) *H. vulgare,* (C) *S. cereale,* (D) *A. sativa.*

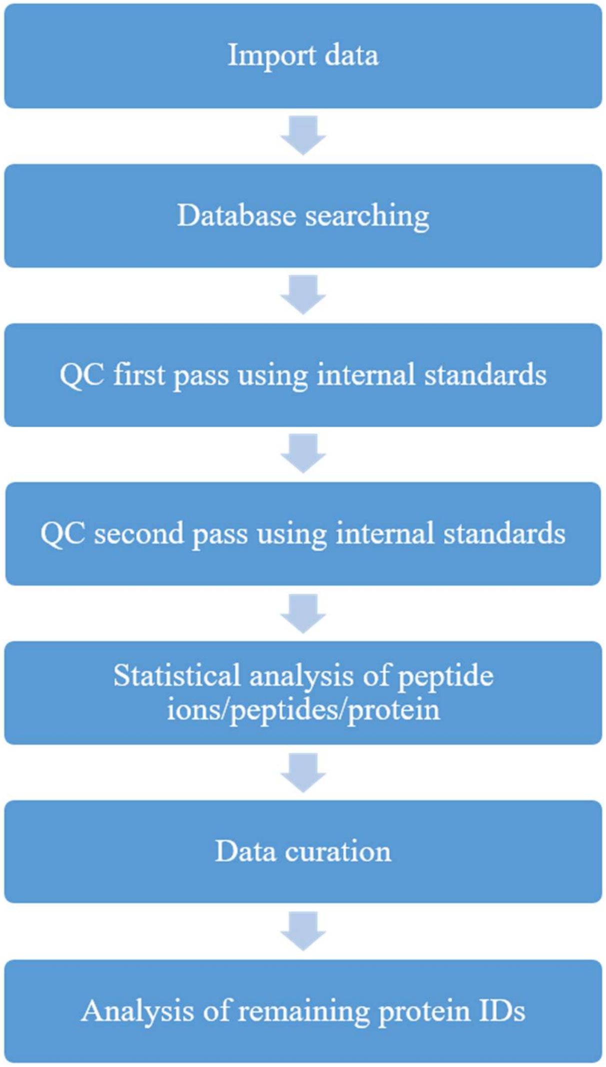
Import data
Database searching
QC first pass using internal standards
QC second pass using internal standards
Statistical analysis of peptide ions/peptides/protein
Data curation
Analysis of remaining protein IDs

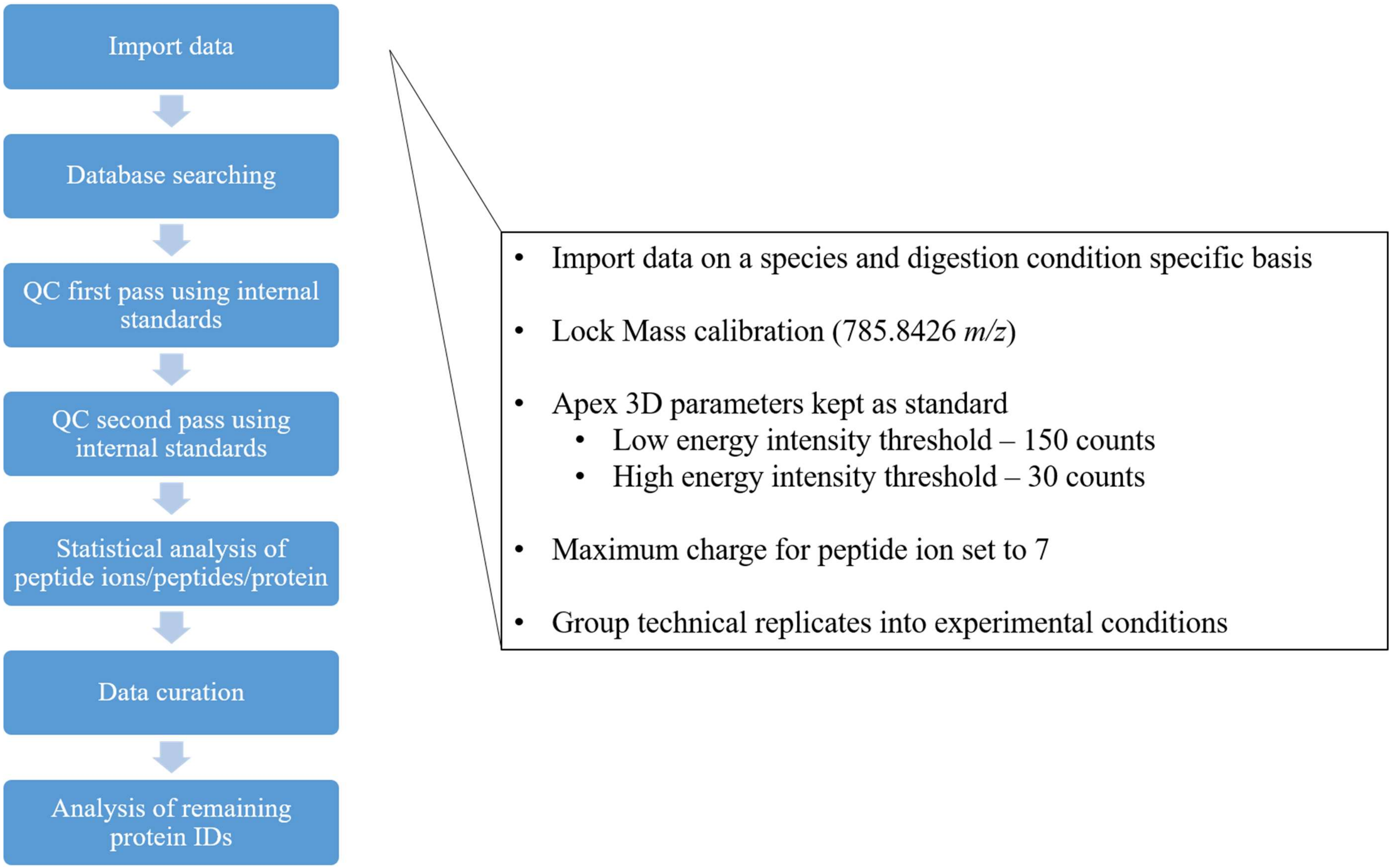
Import data
Database searching
QC first pass using internal standards
QC second pass using internal standards
Statistical analysis of peptide ions/peptides/protein
Data curation
Analysis of remaining protein IDs
• Import data on a species and digestion condition specific basis
• Lock Mass calibration (785.8426 m/z)
• Apex 3D parameters kept as standard
• Low energy intensity threshold – 150 counts
• High energy intensity threshold – 30 counts
• Maximum charge for peptide ion set to 7
• Group technical replicates into experimental conditions

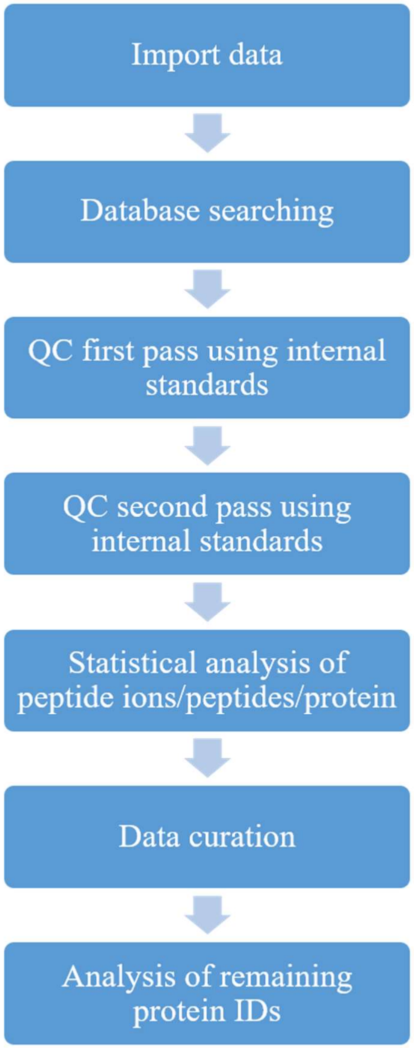

- Protease – Chymotrypsin
  - Cleavage at FYWL
  - Up to 2 missed cleavages

- Fixed mods
  - Carbidomethylation of cysteine
- Variable mods
  - Hydroxylation of proline
  - Oxidation of methionine
  - Deamidation of Q or N
  - N-terminal Pyroglutamic from Q or E

- False discovery rate – 1%

- Mass tolerance
  - Precursor – 10ppm
  - Product – 20ppm

- Fragments per peptide – 1
- Fragments per protein – 3 (ie. one peptide with three fragments or three peptides with one fragment each)
- Peptides per protein – 1 (Has to be unique)

- Search against Viridiplantae downloaded from UniProt

- Append chymotrypsin sequence and cRAP common contaminant database to searchable database as well as GFP and yeast enolase

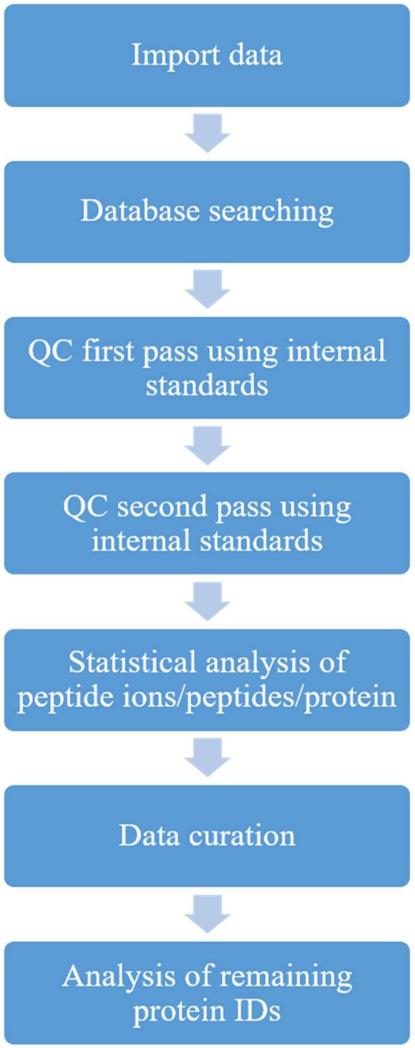

- Archive project
- Remove peptides with score less than 5
- Remove peptides present in <2 technical replicates in all biological replicates
- Using internal standards of enolase from bakers yeast
- Principal component analysis using Progenesis
    - Normalised abundance of peptides
- ISO recommended test for outliers is the Grubbs test
    - $G = |Suspect\ value - \bar{x}|/s$ where s is the standard deviation
    - Critical value of G for n=9 and P=0.05 is 2.215
        - If value >2.215 then it is an outlier and should be rejected
    - Using normalised abundance at peptide level
- Box and whisper plots at peptide level
- CV% of peptide ion abundance between replicates
- Number of peptides for yeast enolase per run
    - Density plots of number of peptides per protein, and length of peptides identified in each run
- Determine if there is any significant difference between the technical replicates or the biological replicates using Grubbs test
    - Remove runs that are significantly different

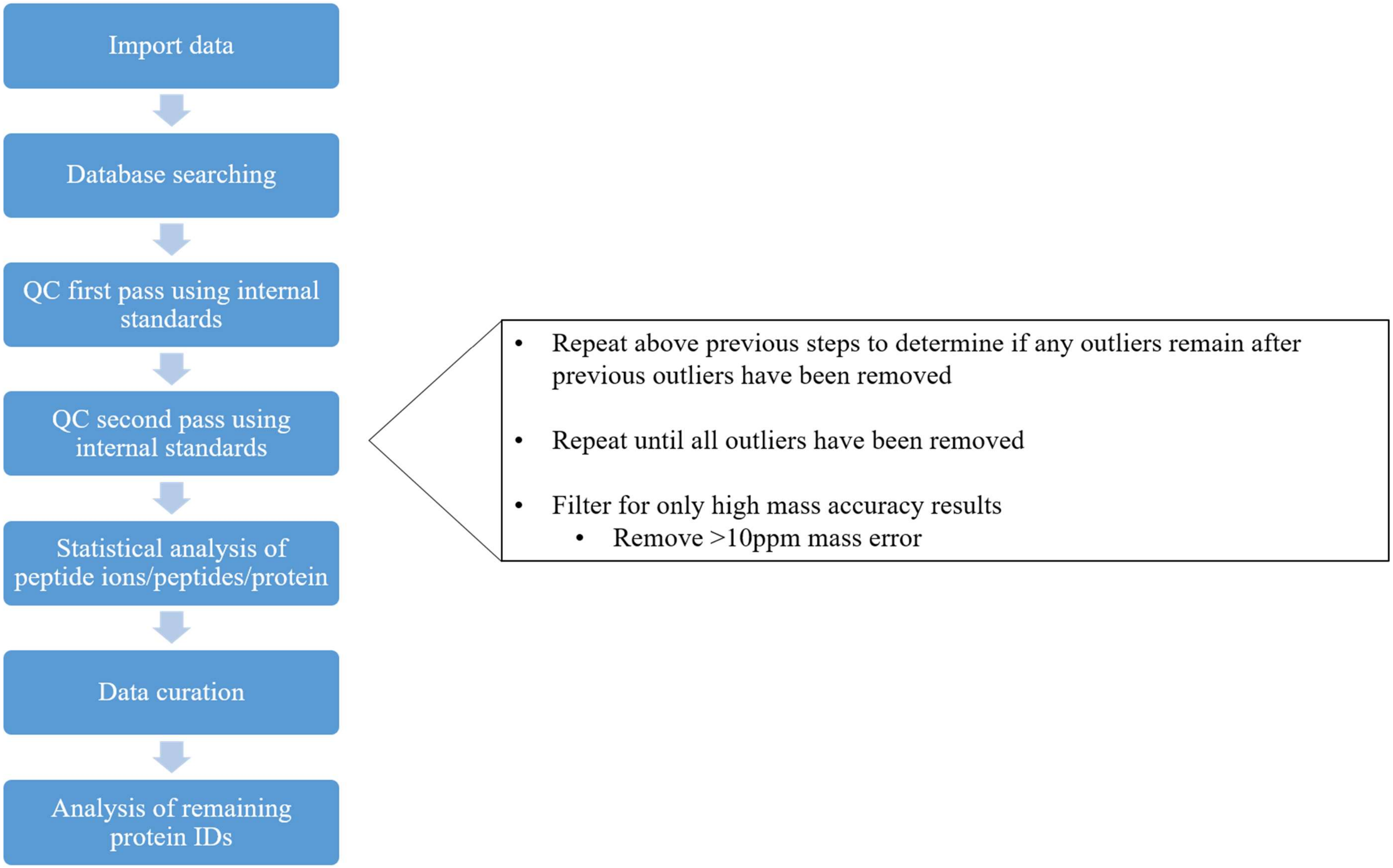
Import data
Database searching
QC first pass using internal standards
QC second pass using internal standards
Statistical analysis of peptide ions/peptides/protein
Data curation
Analysis of remaining protein IDs
• Repeat above previous steps to determine if any outliers remain after previous outliers have been removed
• Repeat until all outliers have been removed
• Filter for only high mass accuracy results
• Remove >10ppm mass error

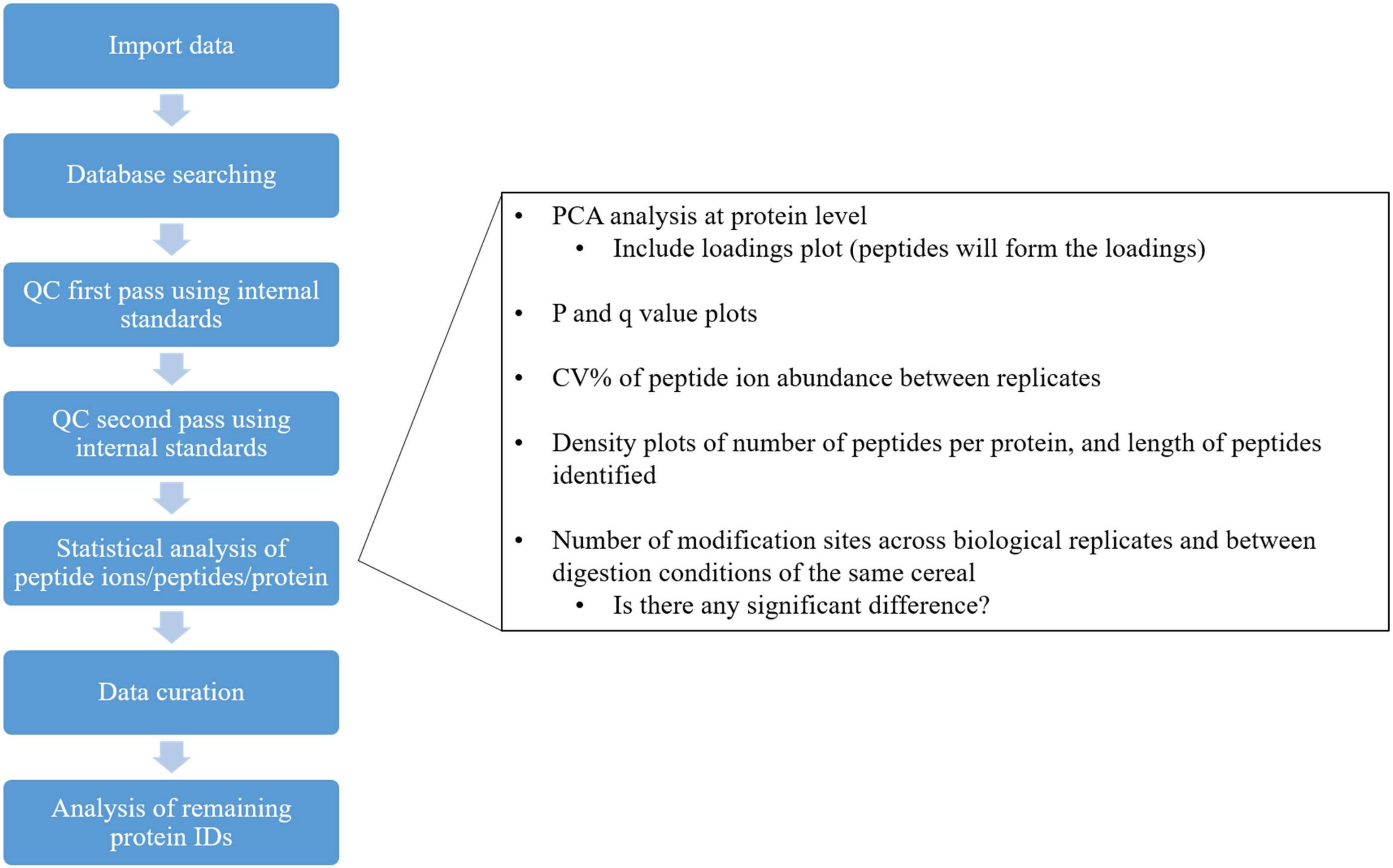
Import data
Database searching
QC first pass using internal standards
QC second pass using internal standards
Statistical analysis of peptide ions/peptides/protein
Data curation
Analysis of remaining protein IDs
PCA analysis at protein level
Include loadings plot (peptides will form the loadings)
P and q value plots
CV% of peptide ion abundance between replicates
Density plots of number of peptides per protein, and length of peptides identified
Number of modification sites across biological replicates and between digestion conditions of the same cereal
Is there any significant difference?

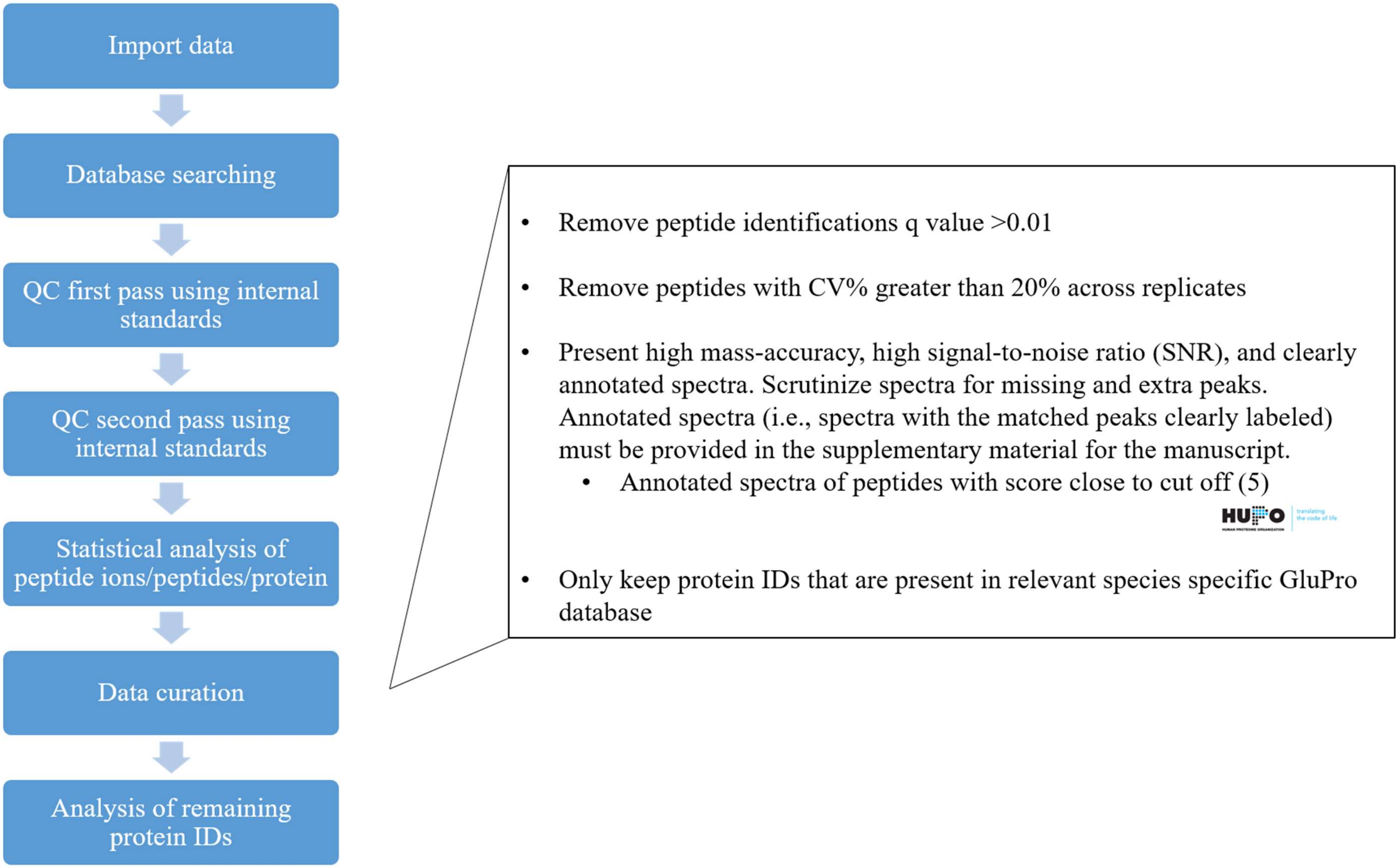
Import data
Database searching
QC first pass using internal standards
QC second pass using internal standards
Statistical analysis of peptide ions/peptides/protein
Data curation
Analysis of remaining protein IDs
Remove peptide identifications q value >0.01
Remove peptides with CV% greater than 20% across replicates
Present high mass-accuracy, high signal-to-noise ratio (SNR), and clearly annotated spectra. Scrutinize spectra for missing and extra peaks. Annotated spectra (i.e., spectra with the matched peaks clearly labeled) must be provided in the supplementary material for the manuscript.
Annotated spectra of peptides with score close to cut off (5)
HUPO
Only keep protein IDs that are present in relevant species specific GluPro database

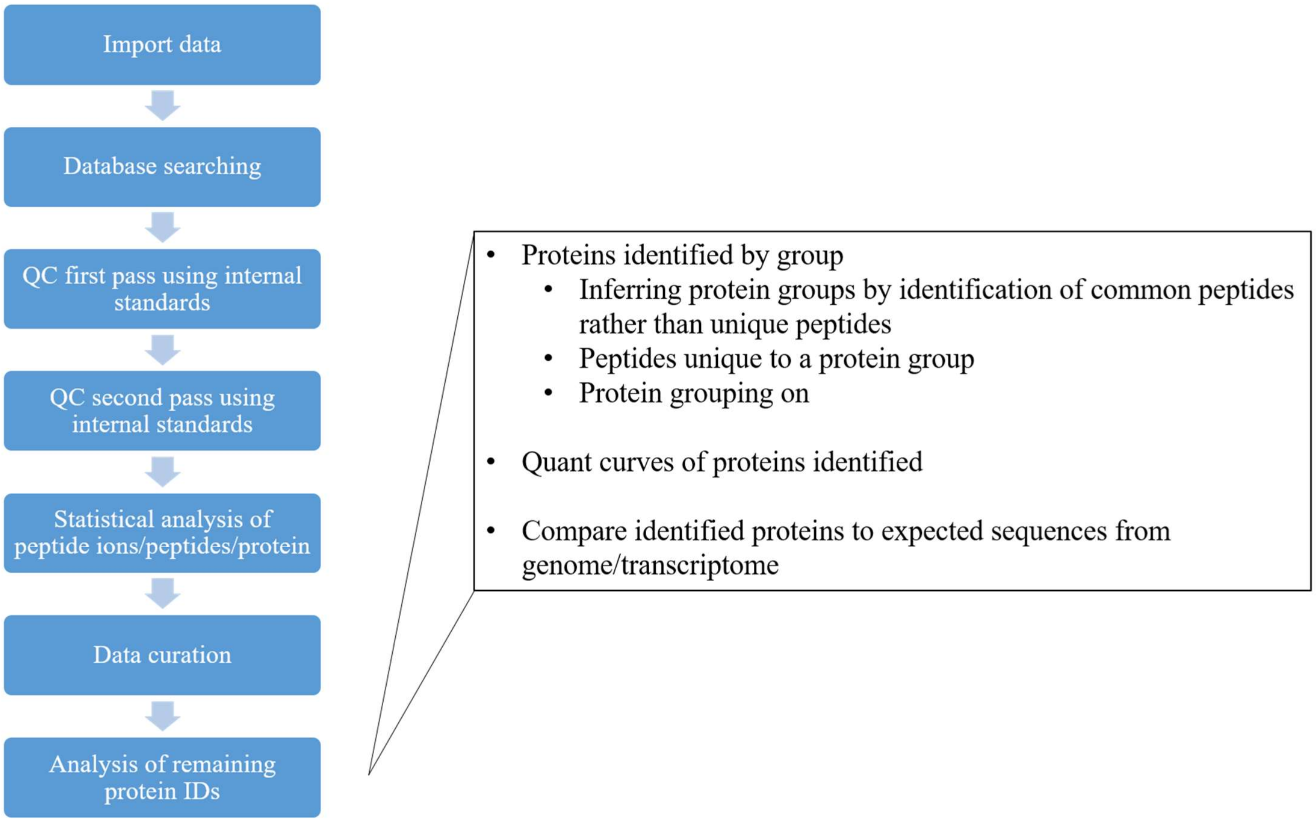

Supplementary Figure S5. Data processing pipeline for analysis of cereals containing gluten using Progenesis QI for Proteomics

A
B

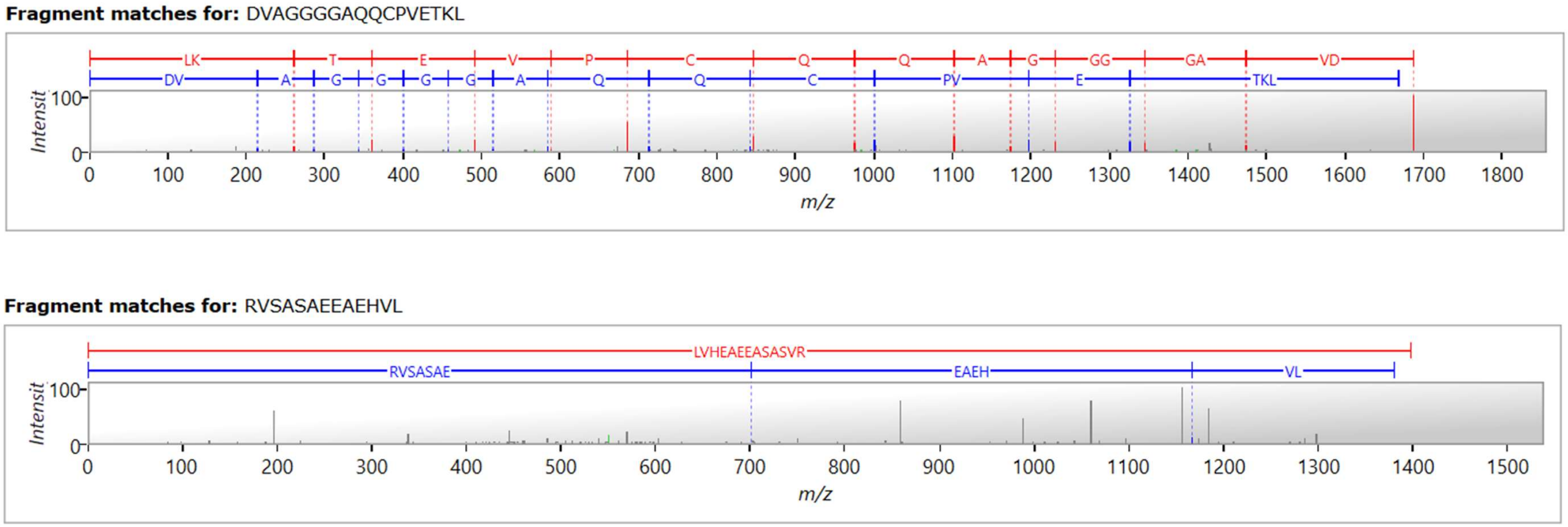

Supplementary Figure S6. Extracted ion chromatograms from two exemplar peptides identified in discovery mass spectrometry.
(A) Peptide with sequence DVAGGGGAQQCPVETKL identified with a score of 9.6443, 36 fragment products and a ppm mass error of 1.85. (B) Peptide with sequence RVSASAEEAEHVL identified with a score of 5.023, five fragment products and a ppm mass error of -4.8470.

The following supplementary data files are available for separate download:

Supplementary Data file 1. Proteins identified from mass spectral data within T. aestivum, H. vulgare, S. cereal and A. sativa samples respectively, using Progenesis QI for Proteomics and protein grouping (XLSX).

Supplementary Data file 2. Peptides identified from mass spectral data within T. aestivum, H. vulgare, S. cereal and A. sativa samples respectively, using Progenesis QI for Proteomics and protein grouping (XLSX).

Supplementary Data file 3. Proteins identified from mass spectral data within T. aestivum, H. vulgare, S. cereal and A. sativa samples respectively, using Progenesis QI for Proteomics and unique peptides only for protein identification (XLSX).

Supplementary Data file 4. Peptides identified from mass spectral data within T. aestivum, H. vulgare, S. cereal and A. sativa samples respectively, using Progenesis QI for Proteomics and unique peptides only for protein identification (XLSX).

Supplementary Data file 5. Principal component analysis loadings used to generate Figure 1 (XLSX).

Supplementary Data file 6. CD-active peptide identified in mass spectral data (XLSX).
